# OsTGA2 confers disease resistance to rice against leaf blight by regulating expression levels of disease related genes via interaction with NH1

**DOI:** 10.1371/journal.pone.0206910

**Published:** 2018-11-16

**Authors:** Seok-Jun Moon, Hee Jin Park, Tae-Heon Kim, Ju-Won Kang, Ji-Yoon Lee, Jun-Hyun Cho, Jong-Hee Lee, Dong-Soo Park, Myung-Ok Byun, Beom-Gi Kim, Dongjin Shin

**Affiliations:** 1 Gene Engineering Division, National Institute of Agricultural Sciences, RDA, Jeonju, Republic of Korea; 2 Department of Biomedical Science and Engineering, Konkuk University, Seoul, South Korea; 3 Institute of Glocal Disease Control, Konkuk University, Seoul, Republic of Korea; 4 Paddy Crop Research Division, National Institute of Crop Science, RDA, Miryang, Republic of Korea; Institute of Genetics and Developmental Biology Chinese Academy of Sciences, CHINA

## Abstract

How plants defend themselves from microbial infection is one of the most critical issues for sustainable crop production. Some TGA transcription factors belonging to bZIP superfamily can regulate disease resistance through NPR1-mediated immunity mechanisms in Arabidopsis. Here, we examined biological roles of OsTGA2 (grouped into the same subclade as Arabidopsis TGAs) in bacterial leaf blight resistance. Transcriptional level of *OsTGA2* was accumulated after treatment with salicylic acid, methyl jasmonate, and *Xathomonas oryzae* pv. Oryzae (*Xoo*), a bacterium causing serious blight of rice. OsTGA2 formed homo- and hetero-dimer with OsTGA3 and OsTGA5 and interacted with rice NPR1 homologs 1 (NH1) in rice. Results of quadruple 9-mer protein-binding microarray analysis indicated that OsTGA2 could bind to TGACGT DNA sequence. Overexpression of *OsTGA2* increased resistance of rice to bacterial leaf blight, although overexpression of *OsTGA3* resulted in disease symptoms similar to wild type plant upon *Xoo* infection. Overexpression of OsTGA2 enhanced the expression of defense related genes containing TGA binding cis-element in the promoter such as AP2/EREBP 129, ERD1, and HOP1. These results suggest that OsTGA2 can directly regulate the expression of defense related genes and increase the resistance of rice against bacterial leaf blight disease.

## Introduction

Plants have developed sophisticated defense mechanisms to overcome a wide variety of phytopathogens including PAMP-triggered immunity (PTI), effector-triggered immunity (ETI), and systemic acquired resistance (SAR) [1, 2]. SAR confers long-lasting protection against a broad spectrum of pathogens involving a small phenolic compound, salicylic acid (SA) [3, 4]. In both local and distal parts of infection region of plant, pathogen infection increases SA and concomitant upregulation of a large number of genes including pathogenesis-related (*PR*) genes, enzymes involved in the synthesis of phytoalexines, and those involved in oxidative stress protection [4]. Exogenous application with SA and its functional analogs such as INA and BTH can induce the expression of *PR* genes and disease resistance [5, 6]. However, overexpression of bacterial salicylate hydroxylase gene *NahG* fails to lead to SA accumulation and expression of *PR* genes [7].

One key component in SA signal transduction is *NON-EXPRESSOR of PR1 (*NPR1) containing a bipartite nuclear localization sequence, an ankyrin-repeat domain, and Broad-complex, tramtrack, Bric-a-brac/Poxvirus, Zinc finger (BTB/POZ) [8, 9, 10]. In unchallenged plant, NPR1 forms a large cytosolic complex via intermolecular disulfide bridges. Upon pathogen infection, SA-triggered redox changes will lead to reduction of NPR1 disulfide bridges. Monomeric NPR1 which is essential for *PR* gene expression then translocates from cytosol to nucleus where NPR1 is phosphorylated [11, 12]. This phosphorylation stimulates turnover through interacting with CUL3-based ubiquitin ligase [13]. Although phosphorylation of NPR1 does not affect its interaction with other transcription factors, phosphorylation and degradation of NPR1 are required for full expression of its target genes such as WRKY38 and WRKY62 [14]. NPR3/NPR4 and NPR1 are reported to function as SA receptors while NPR3/NPR4 act as substrate adaptors for the recruitment of NPR1 to an E3-ubiquitin ligase, leading to its subsequent degradation by the proteasome in Arabidopsis [15, 16]. In addition, transcriptional level of *NPR1* is not changed upon pathogen infection. Instead, NPR1 is regulated at protein levels. However, overexpression of *NPR1* in Arabidopsis can increase resistance to plant pathogens such as *Phytophthora parasitica* and *Pseudomonas syringae* [17]. Arabidopsis NPR1 can interact with rice TGAs. Rice transgenic plants carrying *AtNPR1* overexpression are known to confer tolerance to *Xathomonas oryzae* pv. Oryzae (*Xoo*), a bacterium that causes serious blight of rice [18]. Overexpression of rice *OsNPR1/NH1*, a homolog of Arabidopsis *NPR1* can enhance disease resistance of rice to *Xoo* with highly expressed defense related genes [19].

Plant basic-region leucine-zipper (bZIP) transcription factor super family may form homo- or heterodimers with other bZIP proteins or other classes of transcription factors to positively or negatively regulate the expression of diverse genes involved in differentiation, metabolism, osmotic control, and pathogen defense [20]. Plant bZIP family has 13 groups with 34 Possible Groups of Orthologues (PoGOs) and TGA TFs such as TGA2 and TGA6 in Arabidopsis and TGA2.2 in Tobacco belonging to D subfamily group. They could bind to *activation sequence-1* (*as-1*) found in several pathogenesis-related protein promoters [21–23]. Among D subfamily group of bZIP transcription factors in Arabidopsis, TGA2, TGA5, and TGA6 are required for the establishment of SA-dependent plant defense mechanism through NPR1 interaction [24–26]. TGA2 and TGA3 have strong affinity for NPR1. TGA5 and TGA6 show weak interactions with NPR1 while TGA1 and TGA4 display little or no detectable interaction with NPR1 in yeast [26]. However, *in vivo* TGA1 and TGA4 can bind with NPR1 in Arabidopsis [24]. However, NPR1-1 and NPR1-2 proteins that compromise *PR* gene expression do not interact with TGA2 or TGA3 [24]. To date, 89 bZIP transcription factors have been founded in rice through Genomic survey [23, 27]. Among rice bZIP transcript factors, rTGA2.1functions as a negative regulator in basal defense responses against bacterial *Xanthomonas* infection [28] while several TGA transcription factors including rTGA2.1 and rTGA2.2 can interact with NH proteins both *in vivo* and *in vitro* [29]. However, the functional relationship between NPR and TGA in rice defense mechanism remains unclear. In this study, we choose three bZIP transcription factors belonging to the same subfamily with Arabidopsis TGA2 and rice rTGA2.1 and examined their possible roles in defense response mechanism in rice.

## Materials and methods

### Plant materials and growth conditions

The rice cultivar used in this study was *Oryza sativa* cv. Dongjin. Dehulled rice seeds were surface-sterilized with 70% ethanol and 50% Clorox (Yuhanclorox, Korea) containing Tween 20, washed with distilled water, and grown in 1/2 Murashige and Skoog (MS) medium (supplemented with 1% sucrose, 0.4% phytagel and adjusted to pH 5.8) for 2 weeks under long-day (LD) conditions (16 h light / 8 h darkness). Rice plants were grown from seeds under glasshouse conditions (25±1°C, 60–80% relative humidity).

### Construction of *OsTGA2* overexpressing vector and rice transformation

*OsTGA2* full-length cDNA was cloned into pGA2897 vector including maize ubiquitin promoter [30]. The construct was introduced into *Agrobacterium tumefaciens* LBA4404 *via* electroporation. Transgenic rice was generated using modified early scutellum Agrobacterium-mediated transformation method [31]. Overexpression of transgenic rice was confirmed by semi-quantitative reverse-transcription PCR (RT-PCR).

### Quantitative real-time PCR (qRT-PCR) analysis

Total RNA (5 μg) was extracted from 2-week old plants using RNeasy Plant Mini Kit (Qiagen). It was used for cDNA synthesis using oligo dT and Superscript Reverse Transcriptase first-strand synthesis system (Invitrogen). Quantitative RT-PCR was carried out with gene-specific primers (S1 Table). Gene expression was normalized against the expression of OsActin2 (LOC_Os10g36650). Relative gene expression was analyzed using the ΔΔCt method [32].

### Pathogen inoculation and SA or MeJA treatment

*Xanthomonas oryzae* pv. *oryzae* (*Xoo*) strain KACC10331 was used for pathogen inoculation. Bacterial blight resistance was tested at six-leaf stage of rice plants grown in a growth chamber at 25°C with 80% humidity. Fully opened fifth leaf blades of rice plants were inoculated using the scissor-dip method with absorbance (600 nm) at OD = 0.5 [33]. The length of blight lesions on each leaf blade was measured for each leaf at 14 d post-inoculation. For treatment with SA and methyl jasmonate (MeJA), 2 mM SA or 100 μM JA (Sigma-Aldrich) were sprayed to four-leaf stage rice.

### Rice protoplast isolation and subcellular localization

Protoplasts were isolated from etiolated young seedlings. Surface-sterilized rice seeds were grown on half-strength MS supplemented with 1% sucrose, and 0.4% phytagel and adjusted to pH 5.8 under continuous light conditions for 3 d at 28 °C and transferred to 25 °C dark conditions for 10 d to induce etiolation. Etiolated leaves were then chopped and dipped in K3 enzyme solution [K3 solution with 0.4 M sucrose, 1.5% cellulase R-10 (Yakult Honsa Co., Ltd, Tokyo, Japan), and 0.3% macerozyme R-10 (Yakult Honsha)] supplemented with ampicillin (100 mg l^−1^). This solution was infiltrated via vacuum for 40 min followed by 3–5 h of incubation on a shaking incubator (50 rpm). Chopped tissue with K3 enzyme solution was then filtered via centrifugation through miracloth for 4 min at 300 *g*. An equal volume of W5 solution (154 mM NaCl, 125 mM CaCl_2_, 5 mM KCl, and 2 mM MES, adjusted to pH 5.8) was added to the filtered K3 solution and mixed. Pelleted protoplasts were then resuspended in suspension medium (0.4 M mannitol, 20 mM CaCl_2_, and 5 mM MES, adjusted to pH 5.7). A 10–20 μg aliquot of plasmid DNA carrying *35S*: *GFP-OsTGA2*, *35S*: *GFP-OsTGA3*, or *35S*: *GFP-OsTGA5* was added to this protoplast solution and then mixed with 40% polyethylene glycol (PEG) solution [40% PEG 4000, 0.4 M mannitol, and 100 mM Ca(NO_3_)_2_, adjusted to pH 7.0]. For transfection, the mixture was incubated at 25°C in darkness for 30 min. W3 solution was then added stepwise to dilute PEG solution. After centrifugation (30 g, 10 min), the protoplast pellet was re-suspended in W5 solution and then incubated at 25°C in darkness overnight. Two days after infection, protoplasts were subjected to confocal microscopy (LSM 510 META, Carl Zeiss, Jena, Germany) and GFP signals were detected at excitation wavelength of 488 nm with an argon laser and a capturing emission wavelength of 522 nm for subcellular localization analysis of OsTGAs.

### Bimolecular fluorescence complementation (BiFC) assay

ORFs of *OsTGAs* and *NHs* were fused to cYFP and nYFP, respectively. A pair of constructs as indicated in Figures was transfected using PEG into rice protoplasts. Fluorescence of reconstituted YFP was detected using a confocal laser scanning microscope (LSM 510 META, Carl Zeiss) at excitation wavelength of 515 nm.

### Yeast two-hybrid assay

Yeast two hybrid assays were performed using MATCHMAKER Y2H system (Clontech). Open reading frame (ORF) of each OsTGA and NHs was inserted to yeast two-hybrid vectors PGBKT7 and pGADT7, respectively. Using LiOAC method, each pair of constructs was co-transformed into yeast stain AH109 and plated onto SD minimal media without leucine or tryptophan, or media without leucine, tryptophan, or histidine supplemented with 1 to 10 mM 3-AT (3-amino-1,2,4-trizole) to determine interaction affinity. Images of yeast cells were obtained on the third day.

### Determining putative DNA-binding sequence of OsTGA2

To determine DNA-binding sequence of OsTGA2, a full-length cDNA of *OsTGA2* was inserted into pET32 (a) vector (Novagen) with an N-terminal fusion to a polyhistidine tag and DsRed-monomer fluorescent protein (Novagen). The OsTGA2 fusion protein was purified from *Escherichia coli* strain BL21-CodonPlus (Stratagene). A total of 200 nM of OsTGA2 protein was incubated with Q9 protein-binding microarray including 101,073 features from each 9-mers in phosphate-buffered saline-2% bovine serum albumin, 50 ng/ mL salmon testes DNA (Sigma), and 50 mM zinc acetate at 25°C for 1 h. Fluorescence images were obtained with a 4000B microarray scanner (Molecular Devices). Signal intensities ranged from 0 to 70,000. Significant probes were selected by statistical analysis modified [34]. Probe sequence showing signal intensity of 3,500 from microarray fluorescence image were extracted and analyzed.

### Electrophoretic mobility shift assay (EMSA)

Light Shift Chemiluminescent EMSA Kit (Thermo Fisher Scientific) was used in this experiment. GST-OsTGA2 protein was purified using glutathione-agarose beads (BD biosciences) after incubation. Biotin was labeled at 5’ end of *cis-*element. Biotin-labeled DNA was synthesized. Components of binding reaction included 1X Binding Buffer, 2.5% Glycerol, 5mM MgCl_2_, 50 ng/μL Poly (dI·dC), 0.05% NP-40, 1 Unit Protein, and 20 fmol biotin-labeled DNA. Detailed procedure of EMSA followed the manufacturer’s instructions. Photos were taken using Charge-coupled device (CCD) camera.

### Microarray analysis

For microarray analysis, 2-week-old Dong-jin and OsPYL/RCAR5-OE rice seedlings were grown in MS media. For biological replicates, seedlings were sampled independently three times. Total RNA was extracted using a Mini RNA kit (Qiagen) and analyzed using Rice Gene Expression Microarray and Gene Expression Hybridization kits (Agilent) according to the manufacturer’s instructions. Signals were scanned using an Agilent DNA microarray scanner and signal intensity for individual probes was analyzed using Agilent Feature Extraction Software version 7.5.1. The intensity was normalized by the quantile method and translated into log_2_ scale [35]. These log_2_-normalized intensity data were then uploaded from a tab-delimited text file format to Multi Experiment Viewer (MEV, http://www.tm4.org/mev/) [36] and a heat map was generated. Heat map image based on log_2_-fold-change data in response to drought stress was also created. For drought stress analysis, this work compiled microarray data from ArrayExpress (http://www.ebi.ac.uk/arrayexpress/) and NCBI GEO public microarray database, (http://www.ncbi.nlm.nih.gov/geo/, platform accession number GPL2025, data series accession numbers GSE6901, GSE24048, GSE26280, and E-MEXP-2401: 31 comparisons). Fold-change data under various drought stresses were then integrated with fold-change of OsPYL/RCAR5-OE over Dong-jin (control plant). To identify significant expression patterns, genes having greater or lesser than 2-fold-change with coefficient of variation of less than 1 were chosen.

## Results

### Gene expression of *TGA* transcription factors *OsTGA2*, *OsTGA3*, and *OsTGA5*

TGA transcription factors AtTGA2, AtTGA6, and NtTGA2.2 in Arabidopsis and Tobacco are known to participate in plant defense responses while rTGA2.1/OsbZIP63 functions in basal defense responses of rice as negative regulator [22–25]. However, biological functions of other TGA transcription factors in rice remain unclear. To examine functions of other TGA transcription factors in rice defense response, we first performed phylogenetic analysis with Possible Groups of Orthologues (PoGO) D5 subgroup of bZIP superfamily. Four rice bZIP transcription factors were divided with AtTGA2 and AtTGA5 as TGA transcription factor subclade: OsTGA2/OsbZIP28 (LOC_Os01g59350 / Os01g0808100), OsTGA3/OsbZIP08 (LOC_Os03g20310 / Os03g0318600), OsTGA5/OsbZIP3 (LOC_Os01g17260 / Os01g0279900), and rTGA2.1/OsbZIP63 (S1 Fig). rTGA2.1/OsbZIP63 (LOC_Os07g48820 / Os07g0687700), rTGA2.2 (LOC_Os03g20310 / Os03g0318600) and rTGA2.3 (LOC_Os01g17260.2 / Os01g0279900) share 75, 76 and 78% identity with Arabidopsis TGA2, respectively [33]. OsTGA2, OsTGA3 and OsTGA5 exhibit high homology with rTGA2.1/OsbZIP63. In addition, these transcription factors showed high degrees of conservation within and outside the bZIP domain with NtTGA2 (S2 Fig). Here, we chose three genes (OsTGA2, OsTGA3, and OsTGA5, excluding rTGA2.1) and examined their roles in plant defense responses.

**Fig 1:**
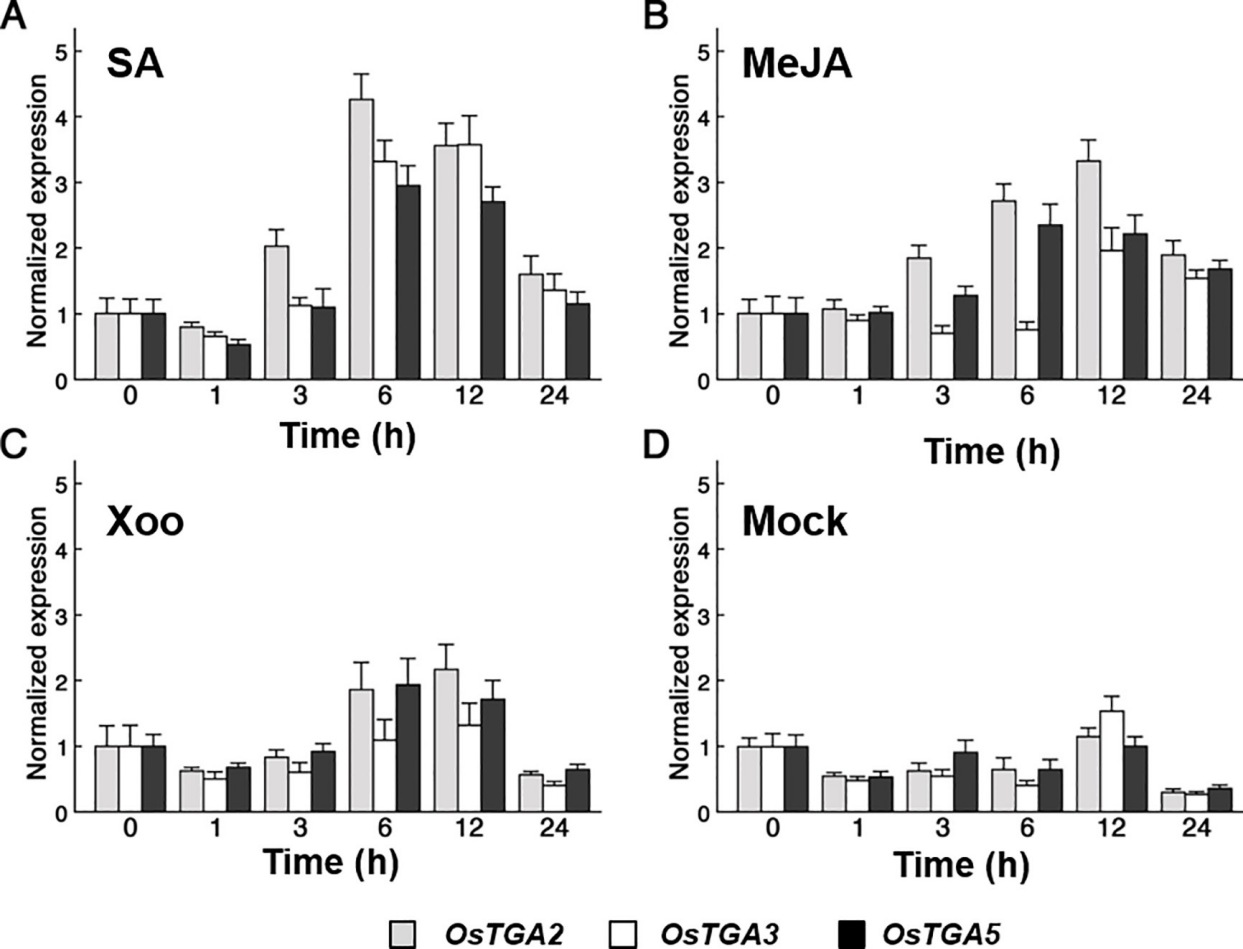
Expression of OsTGA transcription factors in rice. (A and B) Expression profiles of OsTGA genes in rice seedlings in response to treatments of exogenous SA at 2 mM (A) and MeJA at 100 μM (B) (C and D) Expression profiles of OsTGAs upon *Xoo* challenge. Leaves inoculated with *Xanthomonas oryzae* pv. *oryzae* (*Xoo*) KACC10331 (C) or inoculated with sterile water (D) were collected. Total RNA extracted from these samples was used for cDNA synthesis followed by qRT-PCR analysis. Bars represent mean ± standard deviation. Data are obtained from three independent replicated experiments. Gray, white, and black boxes indicate Os*TGA2*, Os*TGA3*, and Os*TGA5*, respectively.

To predict possible roles of these genes in defense responses, we examined their tissue specificity and transcript levels. When tissue specificity of these genes was examined by semi-quantitative RT-PCR using gene-specific primers, *OsTGA2* expressed higher than others in most tissues including leaf blade and leaf sheath while *OsTGA3* and *OsTGA5* showed similar expression patterns, although their expression levels were lower than *OsTGA2* (S4 Fig). When transcriptional levels were checked by real-time PCR analysis with gene-specific primers, transcriptional levels of these genes were slightly oscillated within 0.5 to 1.5-fold under mock treatment conditions. *OsTGA2* transcript was increased in 3 h after salicylic acid (SA) treatment while *OsTGA3* and *OsTGA5* transcripts were increased from 6 h after SA treatment (Fig 1A). Moreover, *OsTGA2* and *OsTGA5* transcripts were enhanced in 3 h and/or 6 h under methyl jasmonate (MeJA) treatment and *Xanthomonas* infection (*Xoo*) (Fig 1B and 1C) whereas *OsTGA3* transcript was not regulated under these conditions (Fig 1). *OsTGA3* whose expression was low in most stages and tissues appeared to respond specifically to SA treatment. These results suggest that *OsTGA2*, *OsTGA3*, and *OsTGA5* might be differently regulated or have different functions in defense responses.

### OsTGAs can bind TGACGT sequence in the nucleus as transcription factors

Sequence analysis indicated that *OsTGA2*, *OsTGA3*, and *OsTGA5* belonged to TGA subclade of bZIPs. To investigate whether they might function as transcription factors, we first examined their subcellular localization in Arabidopsis and rice protoplasts. GFP fused proteins of these three TGA transcription factors were transiently expressed in rice protoplast cells and their subcellular localizations were examined through confocal microscopy (S5 Fig). As putative transcription factors because of their conserved bZIP domains, fluorescent signals were merged with DAPI staining as expected, indicating their localization in the nucleus. This result suggests that these three OsTGAs might have functions in the nucleus as transcription factors to participate in defense responses.

**Fig 2:**
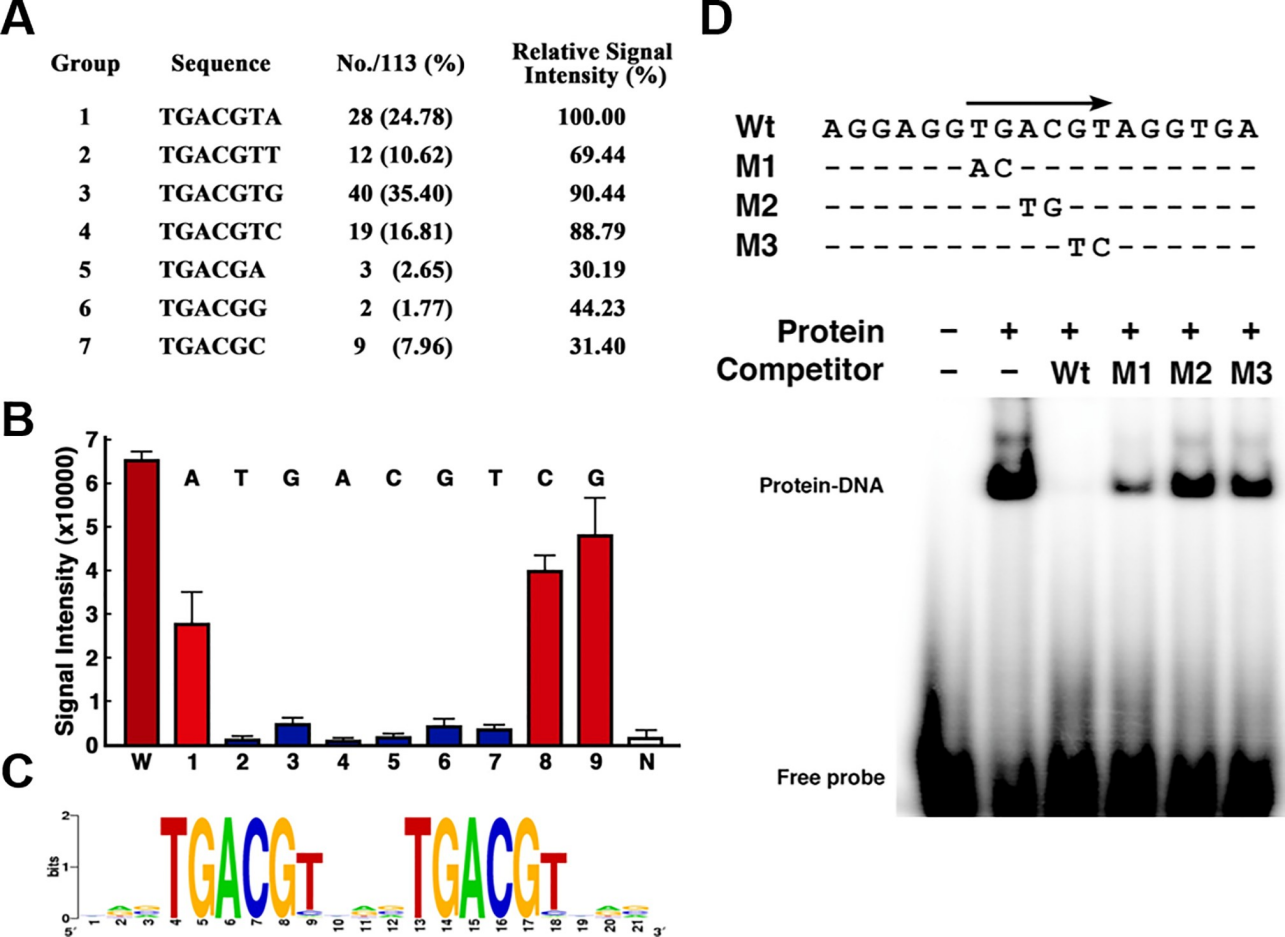
Identification of DNA binding sequences of OsTGA2. (A and B) Identification of DNA-binding sequences of OsTGA2. (A) Summary of putative OsTGA2 DNA binding sequences. Q9 protein-binding microarray analysis was performed using OsTGA2-DsRed fusion protein. A total of 113 probe sequences that produced high signal intensities were selected for further analysis. (B) Signal intensities of TGACGTA sequence and single nucleotide substitution derivatives using Q9 protein-binding microarray. W, Wild-type sequence; 1 to 9, single nucleotide substitution variants (number corresponds to the position of the nucleotide substitution). One probe set that was irrelevant to GGGAAA sequences was used as negative control (N). (C) Sequence logo of TGA binding site based on the information weight matrix model. The height of each letter within a stack is proportional to its frequency at that position in the binding site. Letters are sorted with the most frequent one on the top. The sequence logo was created using WebLogo software (http://weblogo.berkeley.edu). (D) Rice TGA2 binds to *TGACGT* sequences in gel mobility shift assay. Nucleotide sequences of oligonucleotides used as probe and competitors are depicted. Unlabeled wild-type (Wt) and mutated (M1, M2, M3) oligonucleotides were included as competitors.

It has been reported that Arabidopsis and tobacco TGA transcription factors can bind to LS7 and LS5 cis-element of *PR1a* promoter and 35S promoter. To determine whether OsTGAs could bind to such cis-element and determine new DNA binding sequences of OsTGAs, we performed Q9-protein binding microarray with OsTGA2 and obtained 113 putative DNA binding sequences (> 3,500 relative signal intensity). These DNA sequences were segregated into seven groups. Two sequences, TGACGTA and TGACGTG, emerged as predominant DNA binding sequences (Fig 2A). To test whether TGACGT sequence was essential for DNA binding of OsTGA2, we analyzed relative signal intensities of single nucleotide substitution variants of the putative binding sequence. Individual substitutions at all positions of the TGACGT sequence significantly reduced the signal intensity of binding whereas substitutions outside this core sequence had no effect (Fig 2B and 2C). To confirm these results, we performed electrophoresis mobility shift assay. OsTGA2 protein was able to bind to and cause a shift in the mobility of an oligonucleotide containing TGACGT sequence. When wild form of competitor (WT) was added, protein-DNA interaction was completely blocked. However, nucleotide substitution variants (M1, M2 and M3) did not have such effect (Fig 2D). These results suggest that OsTGAs might selectively bind to DNA at TGACGT sequences and function as transcription factors.

### OsTGAs form homo- and hetero-dimer and interact with rice NPR1 homologues

Next, we constructed three OsTGAs in pGBKT7 vector to generate fusion proteins with Gal4 DNA-binding domain and tested their transactivation ability through transactivation assay in yeast. When we examined growth ability of transformed yeast cells on histidine-deficient medium with or without 3-amino-1,2,4-trizole (3-AT), a competitive inhibitor of histidine3 protein, all transformed yeast cells did not grow on histidine-deficient medium with 3-AT (S6 Fig).

**Fig 3:**
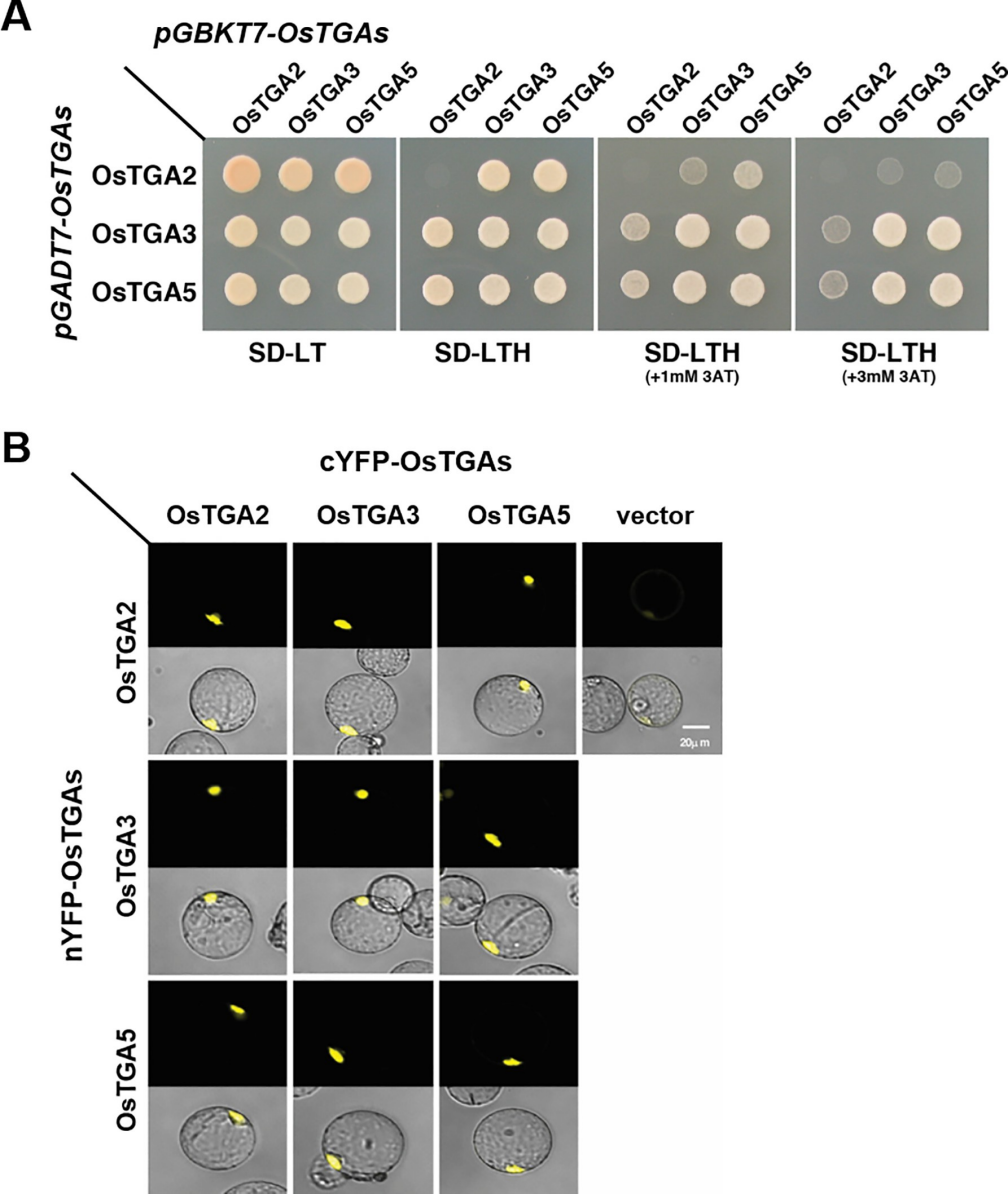
OsTGAs form dimers. (A)Yeast two-hybrid experiments demonstrate dimerization of OsTGAs. OsTGA2, OsTGA3, and OsTGA5 proteins fused to GAL4 DNA-binding domain (BD) and GAL4 activation domain (AD) expressed in yeast stain YH190. Cells were grown on selective media for 3 days at 30°C before pictures were taken. (B) Visualization of protein-protein interactions in rice protoplast by BiFC assay.

Dimerization is required for bZIP transcription factors to be functional in plant [37]. Thus, protein-protein interactions among OsTGA2, OsTGA3, and OsTGA5 were tested in yeast two-hybrid experiment. These three genes were cloned into pGBKT7 and pGADT7 vector as bait and prey, respectively, and transformed into yeast. When we examined the growth ability of transformed yeast cells on histidine-deficient medium with or without 3-AT to investigate homo- or hetero-dimer between OsTGAs in yeast, OsTGA3 and OsTGA5 proteins formed homo- or hetero-dimer. However, OsTGA2 protein was unable to interact with its own protein, although it showed weak associations with OsTGA3 and OsTGA5 proteins (Fig 3A). To further validate this result in plants, we fused these bZIP transcription factors to nYFP and cYFP protein and introduced these constructs into rice protoplast cells and observed yellow fluorescent signals of YFP proteins in rice protoplast. OsTGA3 and OsTGA5 bound with each other as homo- and hetero-dimers in rice protoplast cells. Contrary to yeast two-hybrid results, OsTGA2 protein formed homo-dimer and interacted with OsTGA3 and OsTGA5 proteins in rice protoplast cells (Fig 3B). These results suggest that these OsTGAs might function in different defense mechanisms as homo- and hetero-dimers.

### OsTGAs transcription factors interact with rice NPR1 homologues

**Fig 4:**
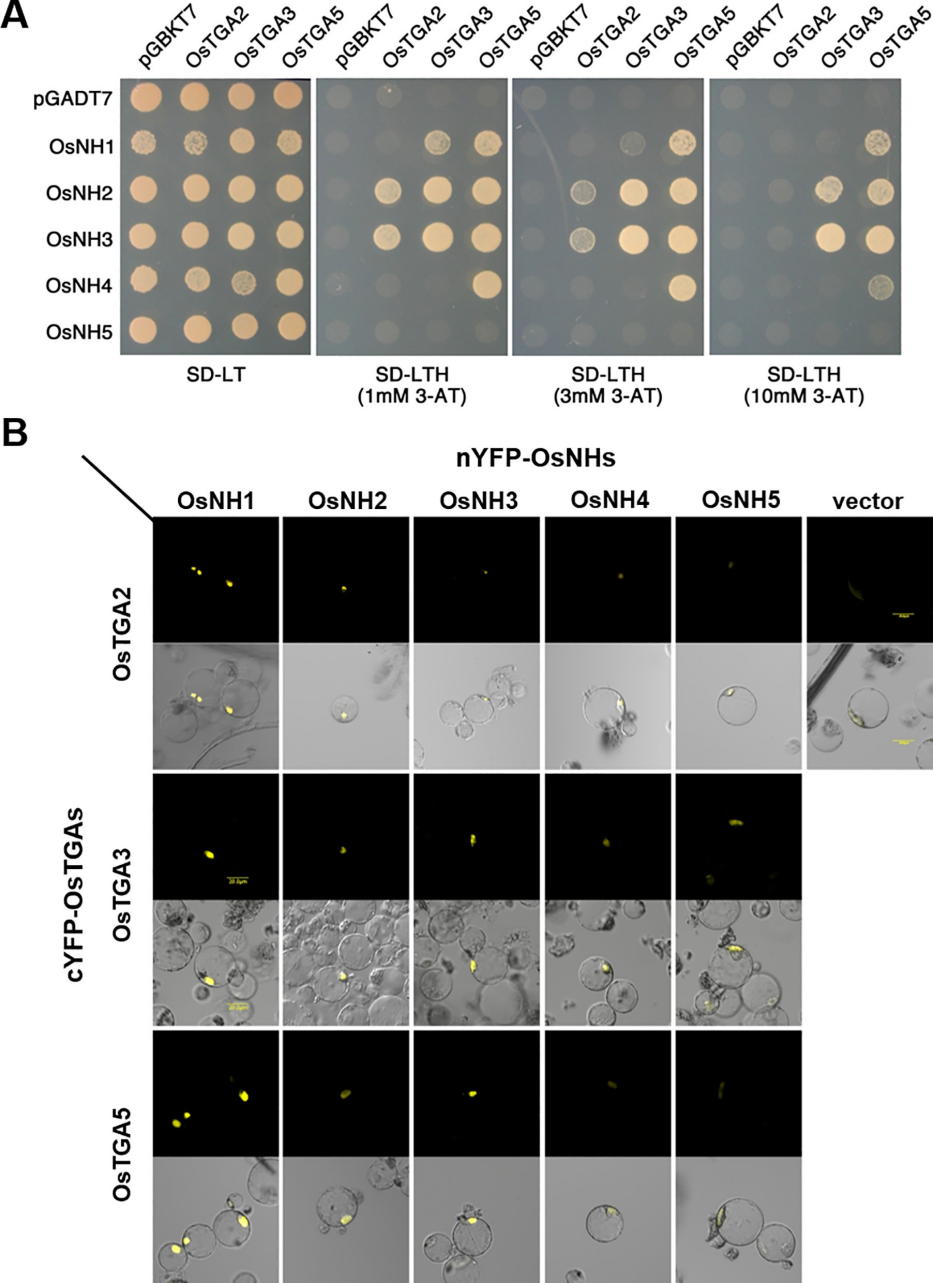
OsTGAs interacts with OsNHs. (A) Interaction of OsTGAs with OsNHs in yeast. OsTGA proteins fused to GAl4-binding domain (BD) were expressed in combination with OsNHs fused to GAL4 activation domain (AD) in yeast stain YH190. Cells were grown on selective media supplemented with 3-AT for 3 days at 30°C before pictures were taken. (B) Visualization of nYFP-OsNHs and cFYP-OsTGAs interactions in rice protoplast by BiFC (Bar is 20 μm).

It has been reported that AtTGA2 and rTGA2.1 are involved in biotic stress response through interaction with NPR1 which regulates the expression of defense-related genes such as *PR1* gene in plants [33, 38]. Thus, we examined whether OsTGAs could interact with rice NPR1-homolog proteins. We selected five NPR1-homolog proteins (NHs) in rice genome (S3 Fig), OsNH1 (Os01g0194300 / LOC_Os01g09800), OsNH2 (Os01g0767900 / LOC_Os01g56200), OsNH3 (Os03g0667100 / LOC_Os03g46440), OsNH4 (Os01g0948900 / LOC_Os01g72020), and two duplicated OsNH5 (Os12g0138500 / LOC_Os12g04410 and Os11g0141900 / LOC_Os11g04600) [5] and performed yeast two-hybrid assay using OsbZIPs as bait and NHs as pray. As shown in Fig 4A, OsTGA2 interacted with NH2 and NH3 on SD-LTH with 1 mM 3-AT. However, such interaction was diminished on SD-LTH with 10 mM 3-AT. OsTGA3 was able to strongly interact with NH2 and NH3. However, OsTGA3 could only bind to NH1 with low affinity while OsTGA5 could bind four NPR1-homolog proteins with different affinities except NH5 protein.

To confirm interactions between OsTGAs and NHs, BiFC assay was performed in rice protoplast. While OsTGA2 did not bind NH1 in the yeast two-hybrid system, it interacted with NH1 and NH2 in the nucleus (Fig 4B). OsTGA3 interacted with all NHs with different interaction strengths. OsTGA5 strongly interacted with NH1 and NH3. Interestingly, NH5 was not associated with any OsTGAs in yeast. It was associated with OsTGA3 brittlely in plant. These results suggest that these three TGA transcription factors can interact with rice NPR1-homolog proteins in the nucleus with different biochemical properties.

### OsTGA2 is involved in resistance against bacterial blight disease

**Fig 5:**
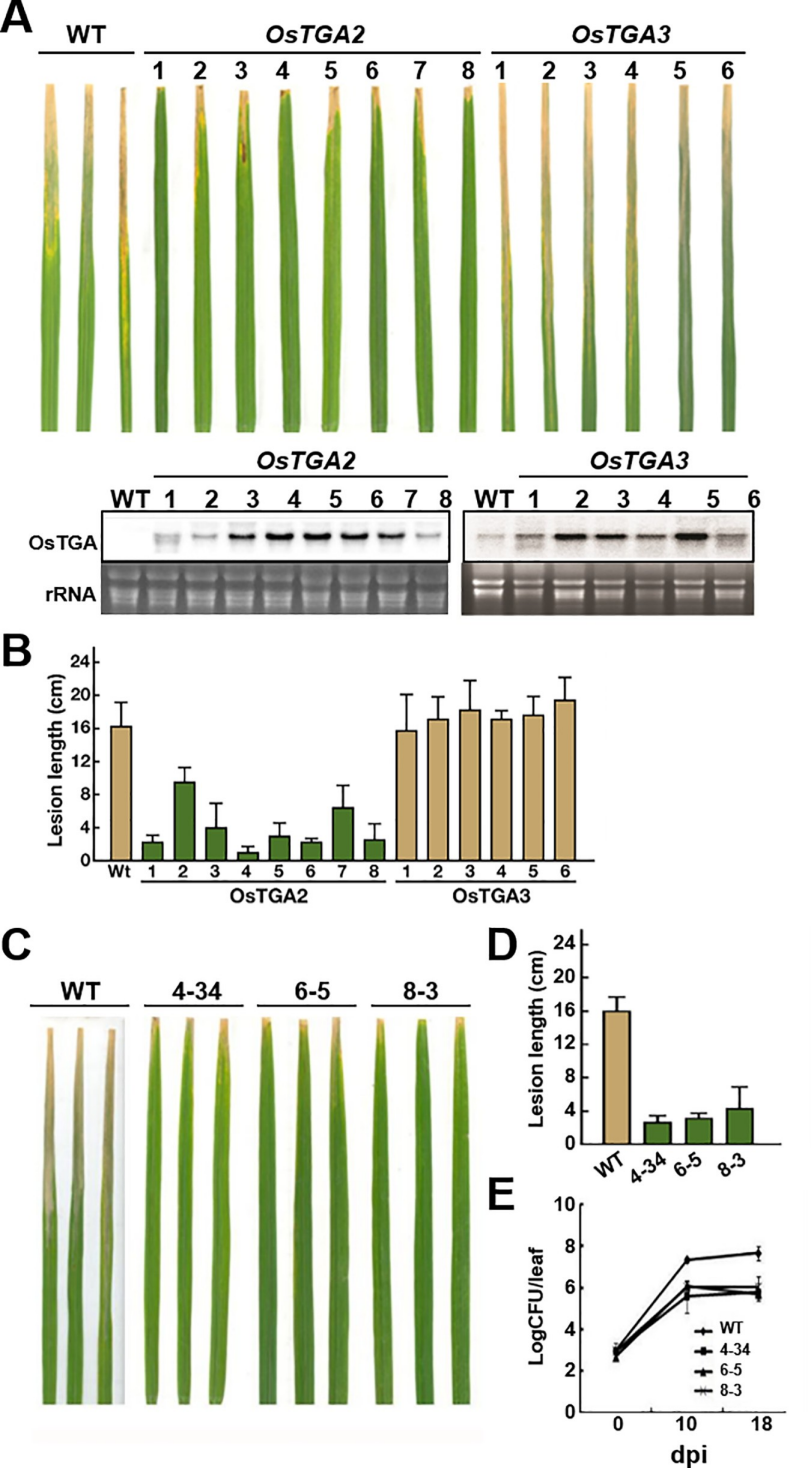
Overexpression of *OsTGA2* confers resistance to rice against bacterial leaf blight. (A and B) *OsTGA2* and *OsTGA3* overexpressing transgenic plants were inoculated with *Xoo* strain KACC10331 when plants were 6-week old. Northern blotting were done to confirm the overexpression in each lines. Three independent experiments were performed and lesion lengths were measure at 3 weeks after inoculation (B). Pictures were taken 3 weeks after inoculation (A). (C-E) Three lines of T3 generation *OsTGA2* overexpressing transgenic plants (4–34, 6–5, 8–3) were infected with *Xoo*. LogCFU/leaf was measured in each line on 10 and 18 day postinoculation (dpi) (E).

Expression analysis and protein-protein interaction results suggested that OsTGA2 and OsTGA3 might have different functions in plant defense responses while OsTGA5 and OsTGA2 might have similar functions. To test this possibility, we generated *OsTGA2* and *OsTGA3* overexpression transgenic plants. Each transgenic plant did not show significant changes in agricultural traits such as height or total seed yield compared with wild-type rice under normal experimental conditions. To test whether OsTGA2 and OsTGA3 might have a role in disease resistance, we inoculated *Xoo* to overexpression plants at T2 generation. Lesion length in *OsTGA2* transgenic plants was considerably shorter (about 4 cm). However, *OsTGA3* transgenic plants did not show any difference in lesion length compared to wild-type plant (Fig 5A and 5B). To further validate their roles in disease resistance, we tested the phenotype after *Xoo* inoculation at T3 generation. As shown in Fig 5C and 5D, *OsTGA2* overexpression lines showed strong resistance against bacterial leaf blight. To test whether the reduced disease symptom in *OsTGA2* transgenic plants was correlated with bacterial multiplication, we checked bacterial growth after *Xoo* inoculation. Bacterial growth of leaves in *OsTGA2* overexpression was about 4.3 × 10^6^ CFU/ml whereas that in wild type was 1.7 × 10^8^ CFU/ml (Fig 5E). This indicates that *OsTGA2*, not *OsTGA3*, can specifically lead to resistance against bacterial leaf blight disease.

### OsTGA2 up-regulates the expression of SA-responsive genes

To determine whether the resistant phenotype of *OsTGA2* transgenic plants was due to higher expression of SA-responsive genes, gene expression patterns in *OsTGA2* overexpression were examined through microarray analysis. When 2 mM SA was used to treat wild type plant for 3 h, 3,196 genes and 1,726 genes were up- and down-regulated 2-fold or more, respectively, compared to those under normal growth conditions. Among upregulated genes in wild type after SA treatment, 252 genes and 63 genes were up-regulated and down-regulated in *OsTGA*2 transgenic rice with SA treatment, respectively (S6 Fig). Notably, transcript levels of several genes including β-1,3-glucanase, glutathione S-transferases, *WRKY45*, and *PR1a* involved in plant defense mechanism were dramatically up-regulated after SA treatment (Table 1). *PR1a* (AF251277, Os07g0129200 / LOC_Os07g03710) was increased about 2.4-fold in *OsTGA2* overexpression and about 7.2-fold after SA treatment in *OsTGA2* overexpression.

**Table 1:** List of up-regulated and defense-related genes in *OsTGA2* over-expression transgenic rice based on microarray analysis.

SEQ_ID	SA treatment	OsTGA2ox	OsTGA2 ox with SA	Gene information
AK065008	4.99	2.54	4.12	Similar to RAV-like protein.
AK109125	5.01	2.17	3.76	Similar to Blind.
AK060078	4.79	2.48	4.43	Universal stress protein (Usp) family protein.
AK070914	3.07	2.11	2.54	Universal stress protein (Usp) family protein.
AK064825	3.15	2.63	4.83	Peptidylprolyl isomerase, FKBP-type domain containing protein.
AK068247	3.32	4.67	8.13	Similar to Beta-1,3-glucanase precursor.
AK121363	2.48	2.48	3.18	Glutathione S-transferase, N-terminal domain containing protein.
AY166599	2.09	2.23	2.69	ATP-dependent Clp protease ATP-binding subunit
AK070706	5.92	2.89	10.35	Conserved hypothetical protein.
J065193L04	6.15	7.48	11.70	NA
AK067235	6.70	3.65	6.76	Oligopeptide transporter OPT superfamily protein.
AK062771	4.39	1.42	8.79	Conserved hypothetical protein.
AK066255	2.68	4.76	5.33	Similar to WRKY transcription factor 45.
AK068486	3.00	3.02	3.38	U box domain containing protein.
AK100029	3.25	3.24	4.22	SAM dependent carboxyl methyltransferase family protein.
J065121D20	3.22	3.49	5.34	Similar to benzyl alcohol benzoyl transferase
AF251277	4.81	2.42	7.24	PR1a protein.
AK107239	3.41	2.35	5.75	UDP-glucuronosyl/UDP-glucosyltransferase family protein.
AK073202	3.87	2.24	2.59	Peroxidase.
AK068382	5.88	2.03	7.94	Hypothetical protein.
AK069626	3.60	3.15	6.06	Conserved hypothetical protein.
AK108716	2.54	2.69	4.62	Protein of unknown function DUF1262 family protein.
AK099466	5.01	4.32	8.99	Conserved hypothetical protein.
AK071383	3.95	1.85	5.62	Similar to Indole-3-glycerol phosphate synthase.
AK067834	3.48	8.80	18.03	WRKY transcription factor 62.
AK099489	3.16	2.02	2.34	Similar to Glutathione S-transferase GST 23.
AK061135	2.81	2.73	6.55	Protein kinase-like domain containing protein.
AK105773	3.40	2.64	2.78	Cytochrome P450 family protein.
J065031G11	3.10	3.21	4.45	Hypothetical protein.
AK107752	2.14	8.69	10.00	NA
AK063710	3.14	2.49	4.50	AAA ATPase domain containing protein.
AK067865	2.27	2.96	4.70	Calmodulin binding protein-like family protein.
AK106721	3.30	2.33	5.55	Epoxide hydrolase family protein.

**Fig 6:**
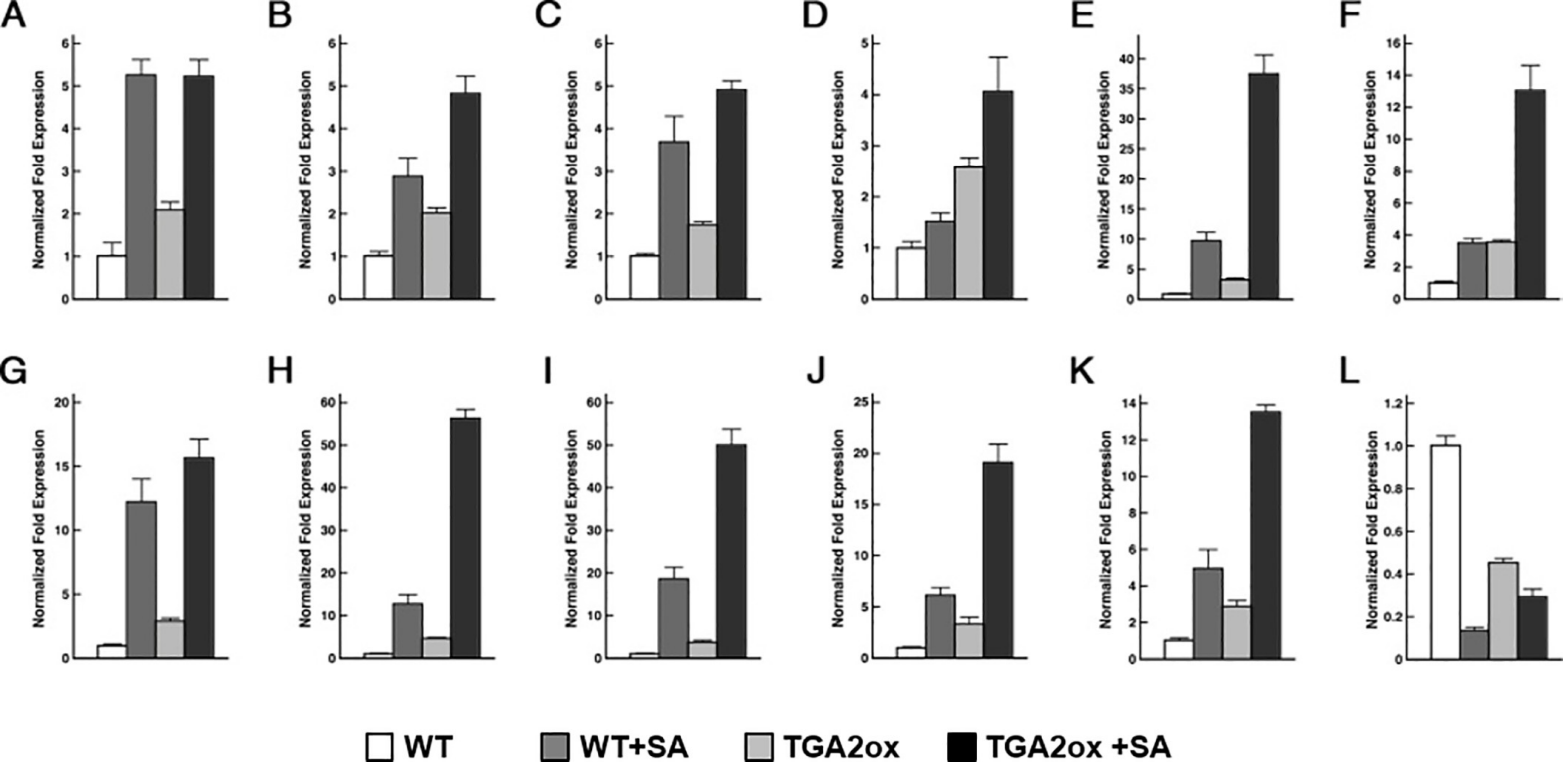
Validation of up-regulated genes using Real-time PCR analysis. Total RNA was extracted from two-week old WT or *OsTGA2* overexpressing transgenic plants treated with 2 mM SA and used for cDNA synthesis followed by Q-RT PCR to check expression levels of stress related genes: (A) AP2/EREBP129, (B) ERD1, (C) indole-3-acetate O-methyltransferase 1-like, jasmonate O-methyltransferase, (D) HSP70-HSP90 organizing protein 1, (E) DEAD-box ATP-dependent RNA helicase 12-like, (F) peptidyl-prolyl cis-trans isomerase FKBP65, (G) YSL13, (H) glycine-rich RNA-binding, (I) Conserved hypothetical protein (Os08g0351300), (J) Conserved hypothetical protein (Os08g0395700), (K) UDP-glucuronosyl/UDP-glucosyltransferase family protein, (L) auxin-induced protein 5NG4.

To validate results of microarray analysis, 12 genes regulated by OsTGA2 were chosen to evaluate their transcript levels through real-time PCR analysis (Fig 6). Expression of AP2/EREBP129 (RAV1-like, AK065008, APETALA2 / ETHYLENE-RESPONSIVE ELEMENT BINDING PROTEIN 129, Os01g0141000 / LOC_Os01g04800) was increased in *OsTGA2* overexpression, although its transcript in wild type plant and *OsTGA2* overexpression showed similar accumulated levels after SA treatment (Fig 6A). EARLY RESPONSIVE TO DEHYDRATION 1 (ERD1, Os02g0526400 / LOC_Os02g32520) (Fig 6B), indole-3-acetate O-methyltransferase 1-like, jasmonate O-methyltransferase (Os06g0323100 / LOC_Os06g21820) (Fig 6C), DEAD-box ATP-dependent RNA helicase 12-like (Os03g0319000 / LOC_Os03g20330) (Fig 6E), YELLOW STRIPE-LIKE GENE 13 (YSL13, Os04g0524500 / LOC_Os04g44300) (Fig 6G), glycine-rich RNA-binding (Os04g0653700 / LOC_Os04g55980) (Fig 6H), two conserved hypothetical proteins (Os08g0351300 / LOC_Os08g26230, and Os08g0395700 / LOC_Os08g30510) (Figs 6I and 6J) and UDP-glucuronosyl/UDP-glucosyltransferase family protein (Os07g0241500 / LOC_Os07g13770) (Fig 6K) exhibited similar patterns: their gene expressions were increased upon SA in wild-type while SA–induced gene expressions were more higher in *OsTGA2* overexpression transgenic plants. In addition, the expression of Hsp70-Hsp90 organizing protein 1 (Os06g0159600 / LOC_Os06g06470) is increased in *OsTGA2* overexpressor (Fig 6D). However, the expression of a gene which is not involved in pathogen defense pathways, auxin-induced protein 5NG4 (Os01g0546400 / LOC_Os01g36580) was is rather decreased upon SA and *TGA2ox* transgenic plants (Fig 6L).

Since OsTGA2 could directly bind to TGACGT cis-element and transcript levels of several genes involved in disease resistance were up-regulated in *OsTGA2* overexpression plant, OsTGA2 might be able to control the expression level of these genes. To test this possibility, we analyzed promoter sequence of up-regulated genes in *OsTGA2* overexpression plant (S7 Fig). When we analyzed promoter DNA sequences of up-regulated genes, there were OsTGA2 binding sequences TGACGT within 2 Kbp of promoter in up-regulated genes such as AP2/EREBP 129, ERD1, and HOP1 suggesting that OsTGA2 might be able to directly control the expression of disease related genes and enhance resistance of rice against bacterial leaf blight disease.

## Discussion

TGA transcription factors belong to bZIP family TFs. They process three domains: an N-terminal domain involved in transactivation activity, a bZIP domain responsible for DNA binding and dimerization, and a C-terminal domain required for interaction with NPR1 [23, 27]. Arabidopsis TGA transcription factors TGA2, TGA5, and TGA6 can positively regulate the expression of *PR1* in systemic acquired resistance [39]. However, Arabidopsis TGA4 acts as a negative regulator for the expression of *PR1a* while TGA1 and TGA4 regulate the expression of nitrate transporter genes [40, 41]. Because NPR1-mediated disease-resistance signaling pathways are similar between Arabidopsis and rice, some rice TGAs must have functions in immune responses. Among rice bZIP transcription factors, OsTGA2, OsTGA3, and OsTGA5 localized in nucleus (S5 Fig) showed the highest sequence identities with AtTGA2, AtTGA6, and NtTGA2.2 in Arabidopsis and tobacco. In this study, we found that the expression of *OsTGA2* was strongly upregulated in immune responses. Thus, OsTGA2 might have different interactions with NPR1-homologs compared to OsTGA3 and OsTGA5 while overexpression of *OsTGA2* might confer resistance to rice against bacterial leaf blight disease by increasing expression levels of disease resistance-related genes.

When plant faces biotic and abiotic stresses, most transcription factors are expressed to regulate downstream functional genes. Arabidopsis *TGA1* and *TGA4* are upregulated in response to nitrate [40]. *OsbZIP1* is induced not only by SA and MeJA, but also by rice blast fungus, *Magnaporthe oryzae* [42]. Rice *OsTFX1* and *OsTGIIAγ1* are induced by *Xoo* infection [43]. It has been reported that 86 transcription factors including *TGA6* and *TGA10* are upregulated as chilling stress response in rice [44]. We found that the transcript level of *OsTGA2* was strongly increased after treatment with SA, MeJA, or *Xoo* (Fig 1). Even though *OsTGA5* transcript level was lower than *OsTGA2*, *OsTGA5* showed similar expression pattern with *OsTGA2*. Therefore, we suggest that *OsTGA2* and *OsTGA5* maybe function defense signaling in rice. However, expression pattern of *OsTGA3* was different with *OsTGA2* and *OsTGA5* under MeJA and Xoo treatment. This result means OsTGA3 has different roles in defense signaling from OsTGA2 and OsTGA5 in rice. In line with these results, when we investigated transcript levels of these genes using public *in silico* database GENEVESTIGATOR [45], *OsTGA2* was mostly expressed in the seedling stage and maturation period of leaf while *OsTGA5* was highly expressed in the booting stage in rice. The expression level of *OsTGA5* was rarely detected during between seedling stage and mature stage (S9 Fig). Bacterial leaf blight disease is caused in mature stage of rice, therefore, we thought OsTGA2 is mainly functions in defense signaling in rice.

Chern et al. [29] have demonstrated that all 3 rice TGA proteins (rTGA2.1, rTGA2.2, and rTGA2.3) can interact with five NH proteins: TGA proteins strongly interact with NH1 and NH3 while the interactions with NH2, NH4, and NH5 are weaker based on the relative strength of β-galactosidase reporter activity in yeast and BiFC assay. When we examined interactions of OsTGA2, OsTGA3, and OsTGA5 with five NH proteins based on the growth strength with different 3-AT concentrations, OsTGA2 could only interact with NH2 and NH3 under low concentration of 3-AT while OsTGA5 showed strong interactions with four NHs except NH5 (Fig 4). In the case of OsTGA3, it interacted NH1, NH2, and NH3, although OsTGA3 had weak interaction with NH1 in yeast. When these interactions were carried out in rice protoplast, Chern et al. have shown that NH1 and NH3 can bind with OsTGAs while NH4 shows weak interactions with OsTGAs. And NH2 showed weak bind property with rTGA2.2 and rTGA2.3, but not TGA2.1 or rLG2 [29]. OsNH1 strongly interacted with all of OsTGAs, and OsNH2 have week bound with OsTGAs. But protein interactions of OsNH4 with OsTGAs were feeble than others (Fig 4). Because they behave quite differently in their interactions with OsNHs and OsTGAs protein, Chern et al. suggested that OsTGAs may function differently in plant [29]. In line with these results, OsTGA2, OsTGA3 and OsTGA5 could form homodimers and heterodimers in rice while OsTGA2 failed to form a homodimer and its interactions with OsTGA3 and OsTGA5 were weaker compared to those in yeast (Fig 4). These interaction differences between OsTGAs and NHs strongly suggest that OsTGA2 might have different roles from OsTGA3 and OsTGA5 in immune response of rice.

Overall from yeast two hybrid and BiFC, OsTGA2 interact with OsTGA1 weakly; OsTGA3 interacts strongly with OsNH3 and weak with OsNH1 and OsNH2. OsTGA5 interact with OsNH1 and OsNH3 strongly but weakly with OsNH2. Actually only OsNH1 and OsNH3 are known to be involved in immune response to *Xoo* by enhancing the resistance to the pathogenic infection [5, 19, 46]. OsNH4 and OsNH5 are rather homologous to BOP1 (BLADE ON PETIOLE1) and BOP2, cytoplasmic and nuclear-localized NPR1 like proteins with BTB/POZ domain and ankyrin repeats and are involved in regulating plant development. [5, 47]. Thus the specific interaction of OsTGA proteins with OsNH1 and OsNH3 support that they function in immunity response.

In line with these results, OsTGA2, OsTGA3 and OsTGA5 could form homodimers and heterodimers in rice while OsTGA2 failed to form a homodimer and its interactions with OsTGA3 and OsTGA5 were weaker compared to those in yeast (Fig 4). These results may be related to protein stability of NH2 in protoplast system which has been suggested by Chern et al. [29]. These interaction differences between OsTGAs and NHs strongly suggest that OsTGA2 might have different roles from OsTGA3 and OsTGA5 in immune response of rice.

*OsTGA2* overexpressing plants in which defense and stress-related genes including β-1,3-glucanase [48], glutathione S-transferase [49], WRKY45 [50], and PR1a [51] genes were up-regulated (Table 1) were more resistant to *Xoo* infection compared to *OsTGA3* overexpressing transgenic plants. The resistance of *OsTGA2* overexpressing lines against pathogen infection was correlated with gene expression levels (Fig 6). It has been reported that *NH1* overexpression exhibits high level of resistance against *Xoo* [19]. Although OsTGA2 did not seem to interact with NH1 in yeast (Fig 4), phytohormone SA enhanced the expression of *OsTGA2* (Fig 1) and OsTGA2 specifically could bind with NH1 (Fig 4). Therefore, OsTGA2 might be involved in NH1-mediated defense pathway in rice.

Among 11 genes upregulated in *TGA2* overexpressing plants in Fig 6, six genes including APETALA2/ETHYLENE-RESPONSIVE ELEMENT BINDING PROTEIN129 (AP2/EREBP129) [52], EARLY RESPONSIVE TO DEHYDRATION1 (ERD1) [53], HSP70-HSP90 organizing protein1 (HOP1), indole-3-acetate O-methyltransferase 1-like/jasmonate O-methyltransferase, and UDP-glucuronosyl/UDP-glucosyltransferase family protein contain a putative TGA2 binding site TGACGT (Fig 3) were found, indicating that these genes could be direct targets of OsTGA2 transcription factor.

Regarding their characterized functions related to biological stress response in plants, ERF5/ERF102 negatively regulates stress response against *A*. *brassicicola* but positively regulates SA signaling and plant defense against bacterial pathogen *Pseudomonas syringae* pv. *tomato* DC3000 [54]. Other ERFs such as ERF1, ORA59, ERF2, and ERF14 appear to regulate several defense-related genes including *PDF1*.*2* and *basic chitinase* in Arabidopsis and increase resistances to plant pathogens *Botrytis cinerea* and *Fusarium oxysporum* [55–59]. Tobacco plants overexpressing OSMOTIN PROMOTER BINDING PROTEIN1 (OPBP1), an AP2/EREBP-like transcription factor, exhibit high expression levels of *PR1a* and *PR5d* with enhanced resistance to *Pseudomonas syringae* pv *tabaci* and *Phytophthora parasitica* var *nicotianae* [60]. Rice transgenic plants overexpressing tobacco *OPBP1* exhibit high tolerance against *Magnaporthe oryzae and Rhizoctonia solani* [61]. Similarly, *TGA2* overexpressing plants showed resistance against blight disease of rice. This might be due to high level of *AP2/EREBP129* which positively regulates defense-related gene expression. EARLY RESPONSIVE TO DEHYDRATION genes are defined as those that are rapidly activated upon dehydration [62]. ERD1 encodes a chloroplast ATP-dependent protease regulatory subunit [61, 62]. ERD1 is down-regulated in flg22 treated *cas* mutant, a loss of function mutant in a chloroplast-localized calcium-sensing receptor (CAS) which is involved in stromal Ca^2+^ transients and responsible for pathogen-associated molecular pattern (PAMP)-induced basal resistance and resistance (R) gene-mediated hypersensitive cell death in Arabidopsis [53]. Thus, upregulated ERD1 in rice *TGA2* overexpressing plants might be involved in pathogen defense.

HOP is a scaffolding protein mediating the interaction of molecular chaperones HSP90 and HSP70. Plant HOP whose expression is increased in response to stress is involved in rice blast resistance by facilitating transport of chitin elicitor receptor kinase1 (CERK1) and inhibiting mitochondrial membrane-based replication of *Carnation Italian ringspot tombusvirus* (CIRV) in tobacco [63, 64]. Furthermore, Arabidopsis HOP3 appears to regulate ER stress response through interacting with Binding Immunoglobulin Protein (BiP), an ER chaperone [65]. Since SA-induced expression of *HOP1* was elevated in *TGA2ox* rice plants (Fig 6C) and the promoter of HOP1 had a putative TGA2 binding site (S8 Fig), TGA2 might participate in broad stress defense pathways.

A putative target of rice TGA2, indole-3-acetate O-methyltransferase 1-like/ jasmonate O-methyltransferase, OsSABATH28 (Os06g0323100 / LOC_Os06g21820) is about 50% identical to Arabidopsis IAA carboxylmethyl transferase 1 (IAMT1) (At5g55250) which regulates leaf pattern, development, and high temperature male sterility [66–68]. Interestingly, *OsSABATH28* is expressed highly in seeds and seeding roots. Thus, it might have other functions.

It is well known that Arabidopsis NPR1 increase defense responsive genes such as pathogenesis-related proteins through interaction with TGA and WRKY transcription factors to enhance bacterial disease resistance caused by *Pseudomonas syringae*. Rice OsNH1 also regulates defense responsive genes and enhance disease resistant caused by *Xanthomonas oryzae*. Rice OsNH1 can bind TGA transcription factors, however, it is still unclear which TGAs function in immune responses. In this study showed that TGA2 could confer pathogen defense response *via* interacting with OsNH1 and regulating the expression of biotic stress mediators. It could serve as a good target for pathogen resistance in crop. The expression of rice Os*TGA2* is increased upon exogenous SA and MeJA treatment and slightly increased upon bacterial infection (Fig 2). It is also expressed in most organs including leaf blade, leaf sheath, internode (Fig 1), and seeds under normal condition, indicating that TGA2 might target other genes not related to plant biotic stress response.

## Supporting information

S1 Fig Phylogenetic tree analysis of bZIP transcription factors in rice based on amino acid sequences. (PDF)

S2 Fig Multiple alignment of OsTGA2, OsTGA3, and OsTGA5 from bZIP containing proteins of Arabidopsis and rice. Sequences were aligned using CLUSTALW and displayed by using BOXSHADE (www.ch.embnet.org/software/BOX_form.html).
(PDF)

S3 Fig Multiple alignment of five NPR1-homolog proteins (NHs) in rice and AtNPR1. Sequences were aligned using CLUSTALW and displayed by using BOXSHADE.
(PDF)

S4 Fig Tissue Comparison of tissue specificity of three TGA transcription factors. Total RNAs from leaf blade, sheath, internode, and seeds were extracted and used for synthesis of cDNA followed by semi-quantitative RT-PCR analysis.
(PDF)

S5 Fig Subcellular localize of OsTGAs in rice and Arabidopsis protoplast. Subcellular localization of GFP-OsTGA tagged proteins transiently expressed in rice (A) and Arabidopsis (B) protoplast. Green fluorescence (GFP), DAPI-staining, and bright-field image were recorded.
(PDF)

S6 Fig Test of transcriptional transactivation properties of OsTGA proteins. *OsTGA* fused with Gal4-DNA binding domain in pGBKT7 vector were transformed to yeast and growth was tested on histidine deficient media with 3-AT.
(PDF)

S7 Fig Microarray analysis of *OsTGA2* overexpressing transgenic plants treated with SA. (PDF)

S8 Fig Promoter analysis of responsive genes. The positions of TGA biding site in promoter of responsive genes are marked with the filled circles in red. The number in parentheses indicates its transcriptional levels in *OsTG*A2 overexpressing transgenic plants.
(PDF)

S9 Fig Heat map comparing expression of OsTGA genes using microarray data available in Genevestigator database. (A and B) Developmental time course of *OsTGA* gene expression in rice. Datasets derived from the analysis of tissue-specific gene expression (A) or developmental time courses (B) of wild-type plants were used for meta-analysis. (C) Expression of *OsTGA*s in wild-type plants treated with different phytohormones and infected with various plant disease.
(PDF)

S1 Table List of primers used in this study. (DOCX)
