# Supplementary material for: OsTGA2 confers disease resistance to rice against leaf blight by regulating expression levels of disease related genes via interaction with NH1
Source: PLoS One. 2018 Nov 16;13(11):e0206910. doi: 10.1371/journal.pone.0206910 (PMC6239283; doi:10.1371/journal.pone.0206910)
Supplement: S1 Fig — (PDF) [file pone.0206910.s001.pdf]

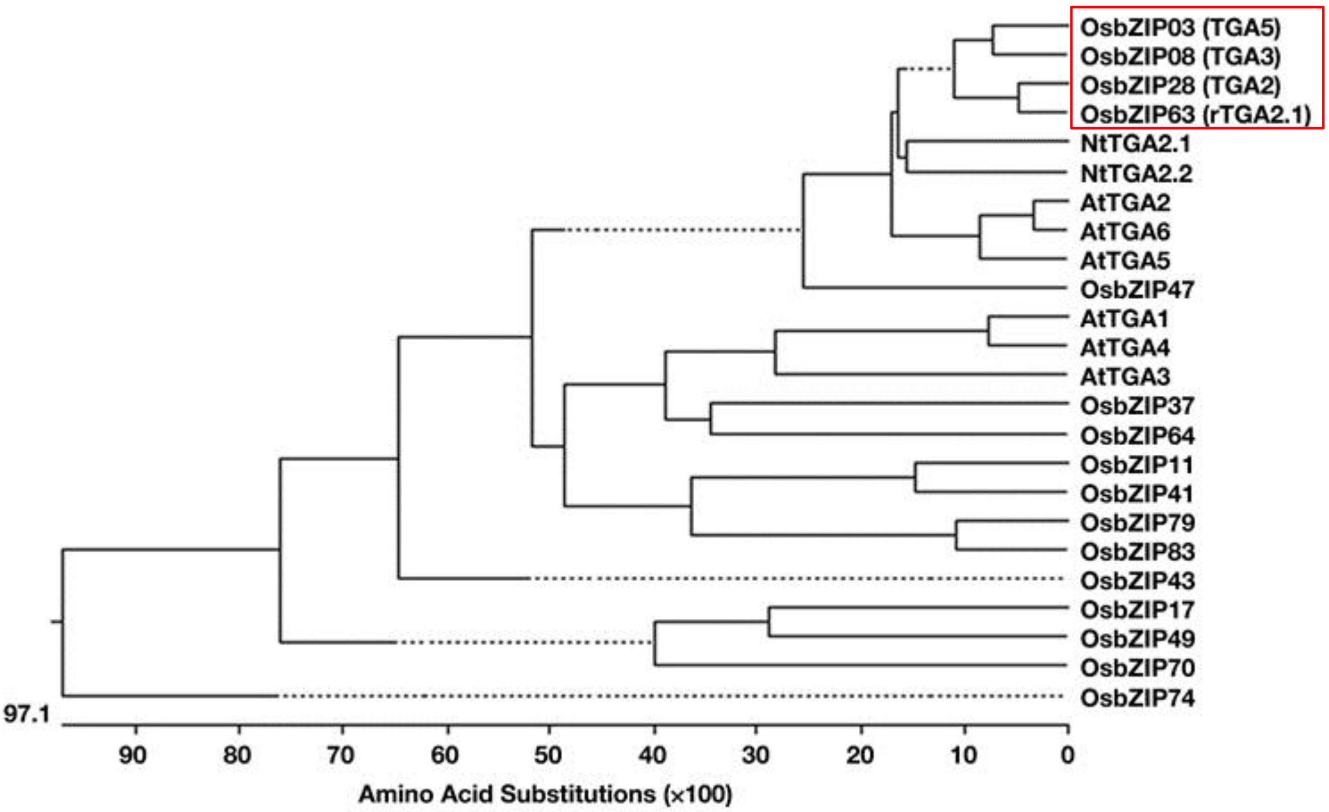

S1 Fig. Phylogenetic tree analysis of bZIP transcription factors in rice based on amino acid sequences
