# Supplementary material for: OsTGA2 confers disease resistance to rice against leaf blight by regulating expression levels of disease related genes via interaction with NH1
Source: PLoS One. 2018 Nov 16;13(11):e0206910. doi: 10.1371/journal.pone.0206910 (PMC6239283; doi:10.1371/journal.pone.0206910)
Supplement: S2 Fig — Sequences were aligned using CLUSTALW and displayed by using BOXSHADE (www.ch.embnet.org/software/BOX_form.html). (PDF) [file pone.0206910.s002.pdf]

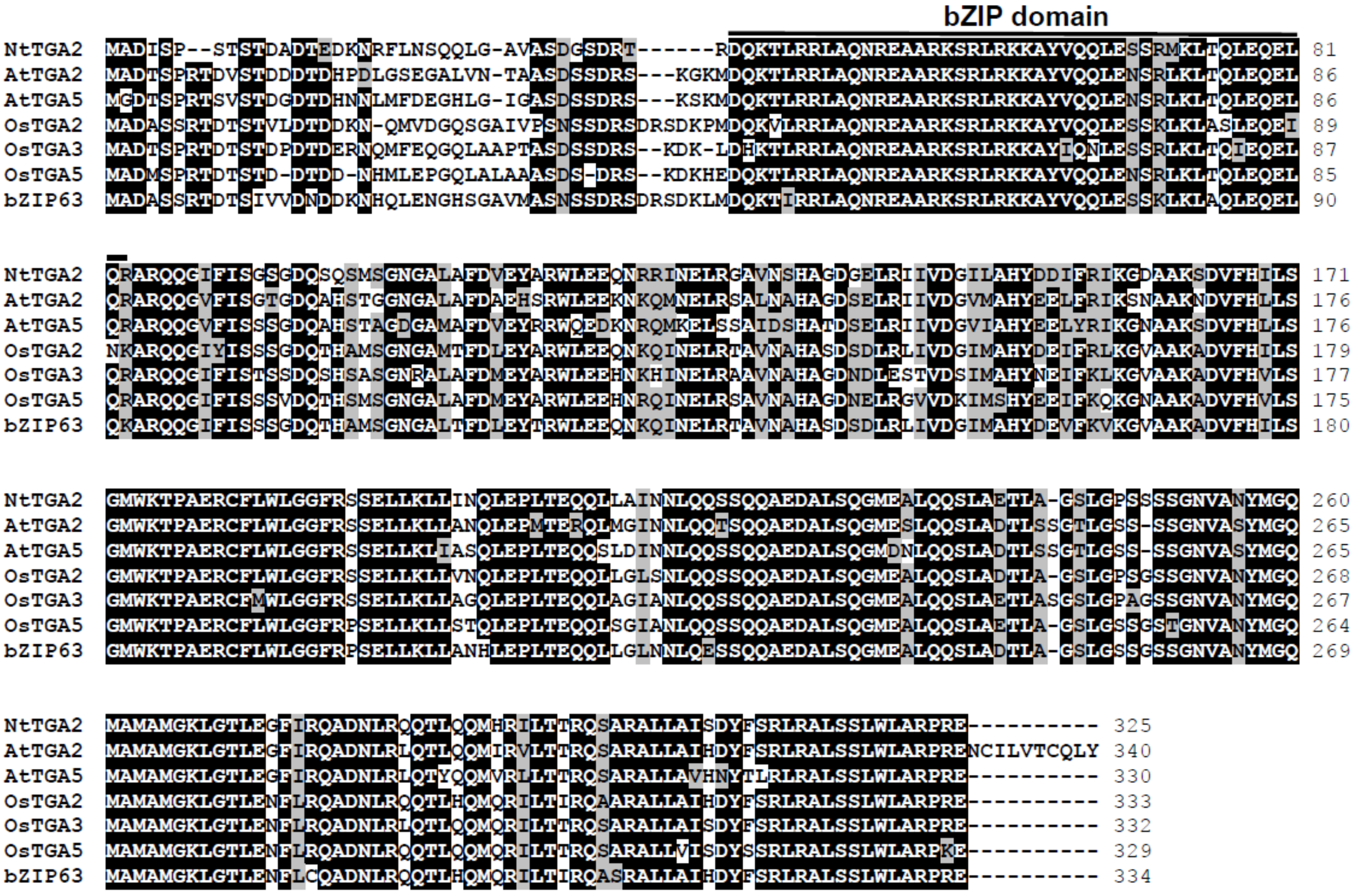

S2 Fig. Multiple alignment of OsTGA2, OsTGA3, and OsTGA5 from bZIP containing proteins of Arabidopsis and rice. Sequences were aligned using CLUSTALW and displayed by using BOXSHADE (www.ch.embnet.org/software/BOX_form.html).
