# Supplementary material for: OsTGA2 confers disease resistance to rice against leaf blight by regulating expression levels of disease related genes via interaction with NH1
Source: PLoS One. 2018 Nov 16;13(11):e0206910. doi: 10.1371/journal.pone.0206910 (PMC6239283; doi:10.1371/journal.pone.0206910)
Supplement: S3 Fig — Sequences were aligned using CLUSTALW and displayed by using BOXSHADE. (PDF) [file pone.0206910.s003.pdf]

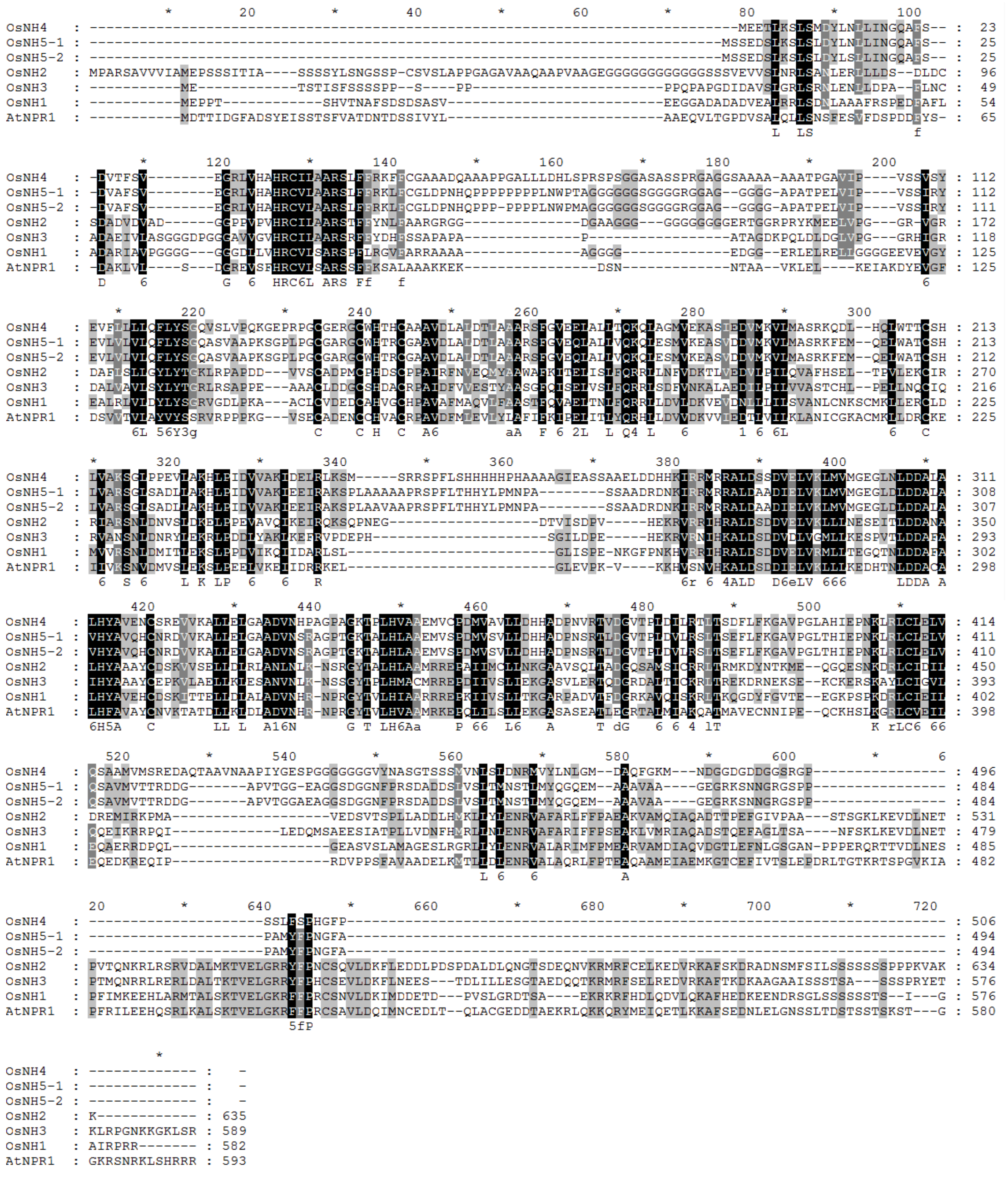

S3 Fig. Multiple alignment of five NPR1-homolog proteins (NHs) in rice and AtNPR1. Sequences were aligned using CLUSTALW and displayed by using BOXSHADE (www.ch.embnet.org/software/BOX_form.html).
