# Supplementary material for: OsTGA2 confers disease resistance to rice against leaf blight by regulating expression levels of disease related genes via interaction with NH1
Source: PLoS One. 2018 Nov 16;13(11):e0206910. doi: 10.1371/journal.pone.0206910 (PMC6239283; doi:10.1371/journal.pone.0206910)
Supplement: S4 Fig — Total RNAs from leaf blade, sheath, internode, and seeds were extracted and used for synthesis of cDNA followed by semi-quantitative RT-PCR analysis. (PDF) [file pone.0206910.s004.pdf]

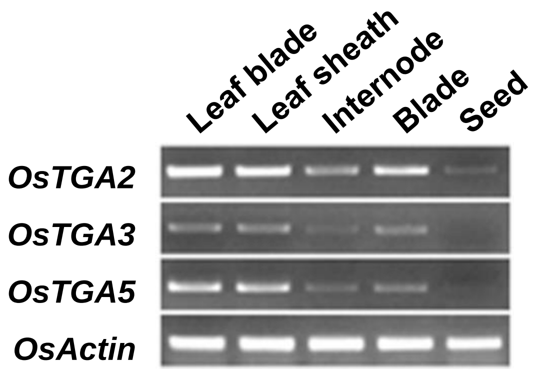

S4 Fig. Tissue Comparison of tissue specificity of three TGA transcription factors.
Total RNAs from leaf blade, sheath, internode, and seeds were extracted and used for synthesis of cDNA followed by semi-quantitative RT-PCR analysis.
