# Supplementary material for: OsTGA2 confers disease resistance to rice against leaf blight by regulating expression levels of disease related genes via interaction with NH1
Source: PLoS One. 2018 Nov 16;13(11):e0206910. doi: 10.1371/journal.pone.0206910 (PMC6239283; doi:10.1371/journal.pone.0206910)
Supplement: S5 Fig — Subcellular localization of GFP-OsTGA tagged proteins transiently expressed in rice (A) and Arabidopsis (B) protoplast. Green fluorescence (GFP), DAPI-staining, and bright-field image were recorded. (PDF) [file pone.0206910.s005.pdf]

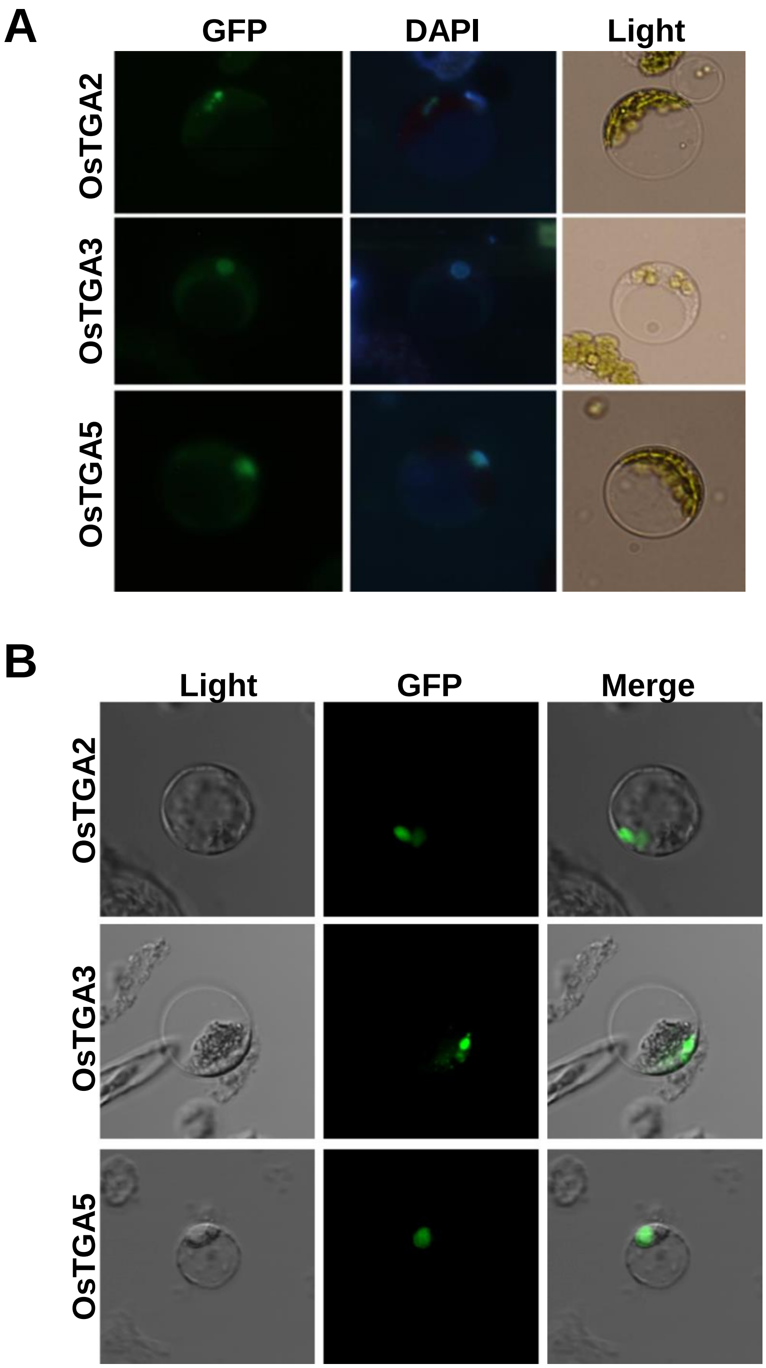

S5 Fig. Subcellular localize of OsTGAs in rice and Arabidopsis protoplast.

Subcellular localization of GFP-OsTGA tagged proteins transiently expressed in rice (A) and Arabidopsis (B) protoplast. Green fluorescence (GFP), DAPI-staining, and bright-field image were recorded.
