# Supplementary material for: OsTGA2 confers disease resistance to rice against leaf blight by regulating expression levels of disease related genes via interaction with NH1
Source: PLoS One. 2018 Nov 16;13(11):e0206910. doi: 10.1371/journal.pone.0206910 (PMC6239283; doi:10.1371/journal.pone.0206910)
Supplement: S6 Fig — OsTGA fused with Gal4-DNA binding domain in pGBKT7 vector were transformed to yeast and growth was tested on histidine deficient media with 3-AT. (PDF) [file pone.0206910.s006.pdf]

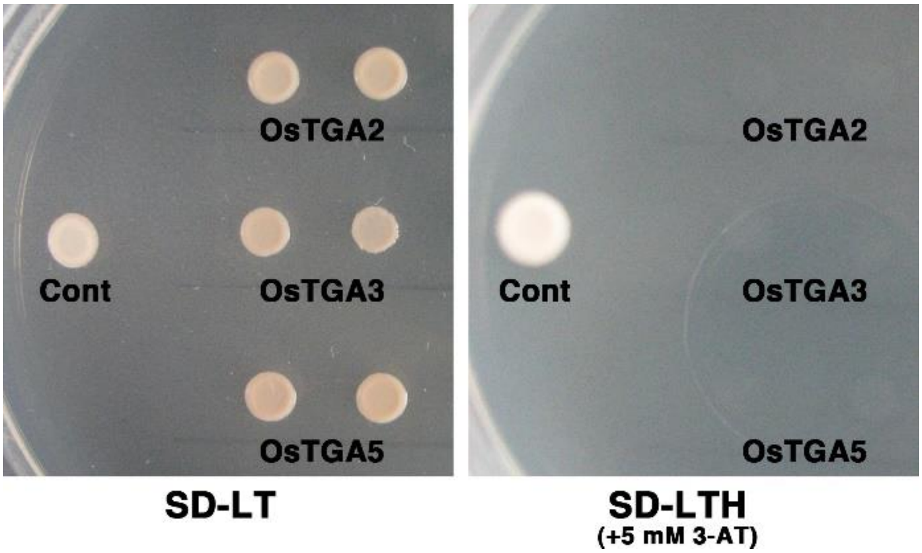

S6 Fig. Test of transcriptional transactivation properties of OsTGA proteins.

OsTGA fused with Gal4-DNA binding domain in pGBKT7 vector were transformed to yeast and growth was tested on histidine deficient media with 5mM 3-AT.
