# Supplementary material for: OsTGA2 confers disease resistance to rice against leaf blight by regulating expression levels of disease related genes via interaction with NH1
Source: PLoS One. 2018 Nov 16;13(11):e0206910. doi: 10.1371/journal.pone.0206910 (PMC6239283; doi:10.1371/journal.pone.0206910)
Supplement: S8 Fig — The positions of TGA biding site in promoter of responsive genes are marked with the filled circles in red. The number in parentheses indicates its transcriptional levels in OsTGA2 overexpressing transgenic plants. (PDF) [file pone.0206910.s008.pdf]

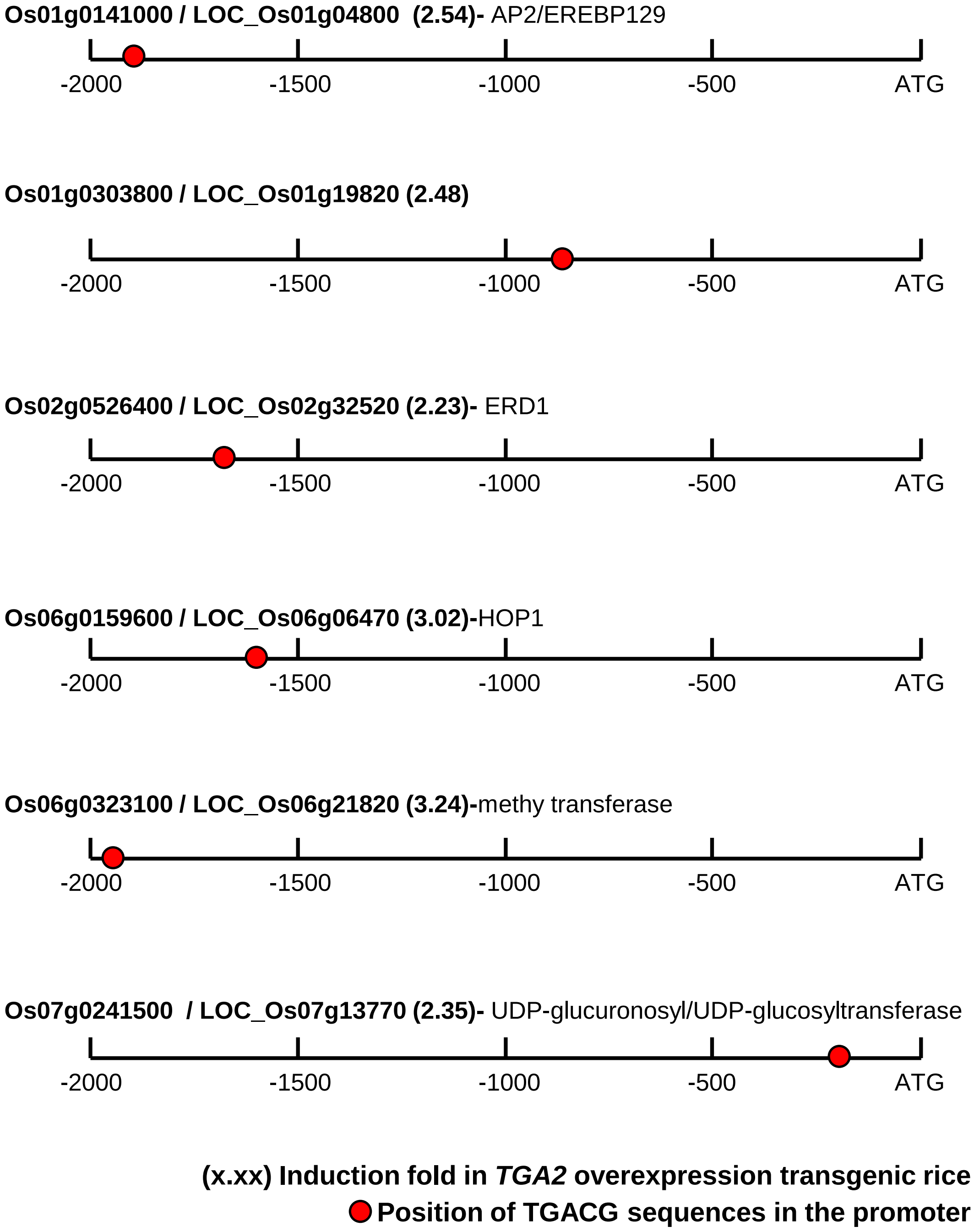

S8 Fig. Promoter analysis of responsive genes. The positions of TGA biding site in promoter of responsive genes are marked with the filled circles in red. The number in parentheses indicates its transcriptional levels in *OsTG*A2 overexpressing transgenic plants.
