# Supplementary material for: OsTGA2 confers disease resistance to rice against leaf blight by regulating expression levels of disease related genes via interaction with NH1
Source: PLoS One. 2018 Nov 16;13(11):e0206910. doi: 10.1371/journal.pone.0206910 (PMC6239283; doi:10.1371/journal.pone.0206910)
Supplement: S9 Fig — (A and B) Developmental time course of OsTGA gene expression in rice. Datasets derived from the analysis of tissue-specific gene expression (A) or developmental time courses (B) of wild-type plants were used for meta-analysis. (C) Expression of OsTGAs in wild-type plants treated with different phytohormones and infected with various plant disease. (PDF) [file pone.0206910.s009.pdf]

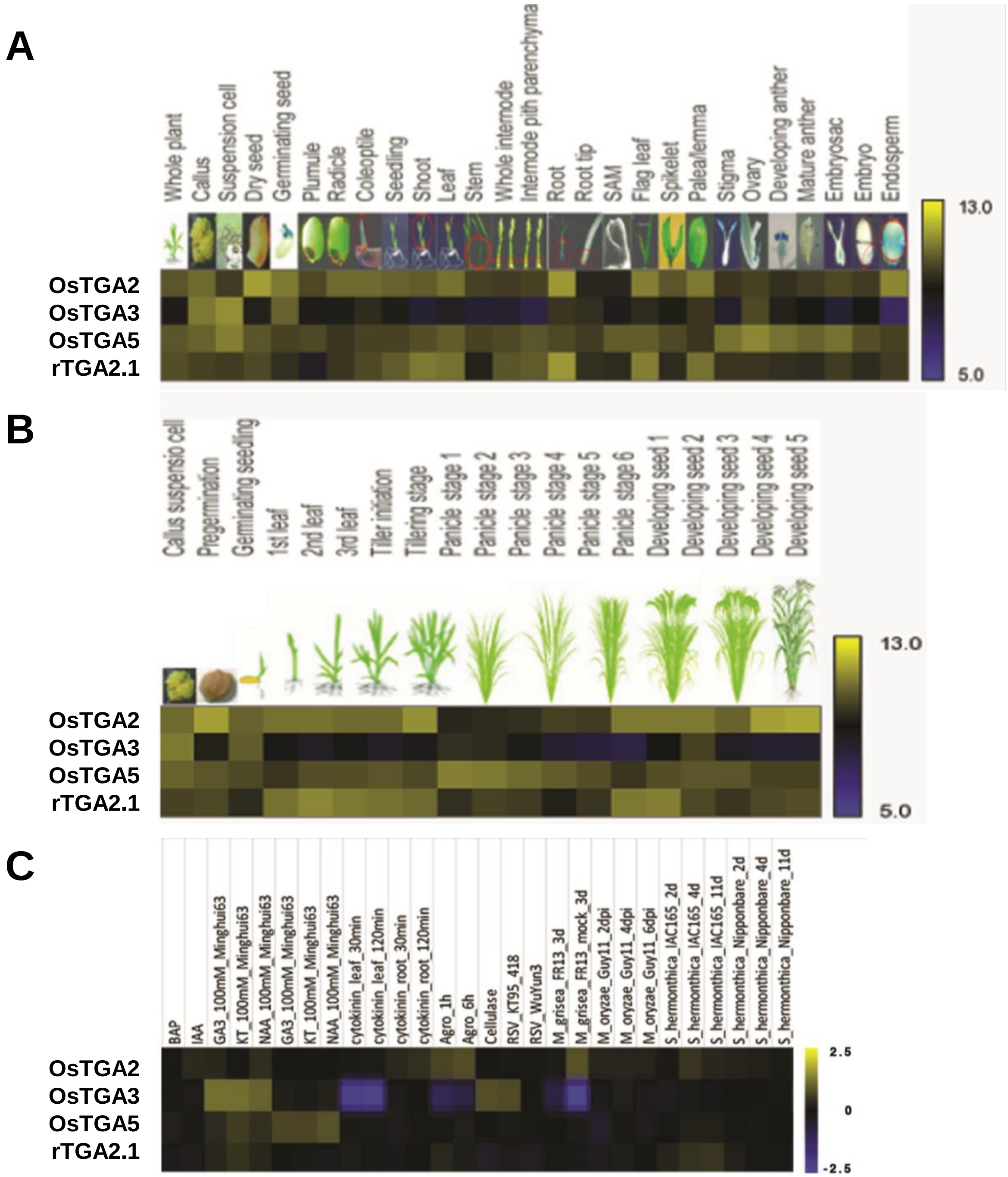

S9 Fig. Heat map comparing expression of OsTGA genes using microarray data available in Genevestigator database.

(A and B) Developmental time course of *OsTGA* gene expression in rice. Datasets derived from the analysis of tissue-specific gene expression (A) or developmental time courses (B) of wild-type plants were used for meta-analysis.

(C) Expression of *OsTGA*s in wild-type plants treated with different phytohormones and infected with various plant disease.
