# Supplementary material for: OsTGA2 confers disease resistance to rice against leaf blight by regulating expression levels of disease related genes via interaction with NH1
Source: PLoS One. 2018 Nov 16;13(11):e0206910. doi: 10.1371/journal.pone.0206910 (PMC6239283; doi:10.1371/journal.pone.0206910)
Supplement: S1 Table — (DOCX) [file pone.0206910.s010.docx]

**S1 Table. List of primers used in this study**

| Primer name | sequence | purpose |
| --- | --- | --- |
| OsTGA2_F | ATGGCTGATACAAGTCCAAGGACTG | cloning |
| OsTGA2_R | TTATTCCCTTGGACGGGCGAGCC |  |
| OsTGA3_F | ATGGCAGATGCTAGTTCGAGGAC |  |
| OsTGA3_R | TTACTCCCGTGGCCTAGCAAGC |  |
| OsTGA5_F | ATGGCAGATATGAGCCCTAGGAC |  |
| OsTGA5_R | CTATTCTTTCGGCCGAGCAAGCC |  |
| Os01g0141000_For | GGCGACGAGAGGCCAACCAC | Q-RT-PCR |
| Os01g0141000_Rev | ACAGCAGGGCACCTAGGGCA |  |
| Os02g0526400_For | AAGGCTTTGGCTCGCCGCTT |  |
| Os02g0526400_Rev | GGCCTTGTCAGGGAGCTGCC |  |
| Os06g0323100_For | ACCGTCTGTTCCCGGAGCGA |  |
| Os06g0323100_Rev | GTCGGACTGGAACTGCCGCC |  |
| Os06g0159600_For | ACAGGGACGCGTTTGCGGAG |  |
| Os06g0159600_Rev | AGCTCGACAAGGCATCGAGCT |  |
| Os03g0319000_For | CCCATGCCGTTCCAGAGCGT |  |
| Os03g0319000_Rev | AGCAAGCGTACATGCACAGCA |  |
| Os01g0564300_For | CGTCGTCCTGTCAAGCCGGG |  |
| Os01g0564300_Rev | ACATCGGCGGCAGTCGTTGG |  |
| Os04g0524500_For | TTCTTGCTGGCCTGGCTGCC |  |
| Os04g0524500_Rev | AGACGGCAGCATTTGGCGCT |  |
| Os04g0653700_For | GCGGGGCAGCTCGATCTTGA |  |
| Os04g0653700_Rev | CCCTTGCCATCGGCGCAGTT |  |
| Os08g0351300_For | GACTGCGACTCCGCCGCTAC |  |
| Os08g0351300_Rev | CCAGCCCTTGCTGCTGAGGG |  |
| Os08g0395700_For | AGCAATCGACAGGCCACGCA |  |
| Os08g0395700_Rev | GCTTGGCCGATGTTGCGGC |  |
| Os07g0241500_For | TGGAGAGCAGTGGCCAGCCT |  |
| Os07g0241500_Rev | ACCCTCCCACTGCGTGGTGT |  |
| Os01g0546400_For | TCGCTCTGGCGATTGAGAGGGA |  |
| Os01g0546400_Rev | TGGCCAAAAAGACCGGCCCA |  |
| Actin_For | TGCTATGTACGTCGCCATCCAG |  |
| Actin_Rev | AATGAGTAACCACGCTCCGTCA |  |
| OsTGA2_Y_For | CCCGGGATGGCTGATACAAGTCCAAGG | Y2H |
| OsTGA2_Y_Rev | GGATCCTTATTCCCTTGGACGGGCGAG |  |
| OsTGA3_Y_For | CCCGGGATGGCTGATACAAGTCCAAG |  |
| OsTGA3_Y_Rev | GGATCCTCATGGCTGATACAAGTCCAAG |  |
| OsTGA5_Y_For | CCCGGGATGGCAGATATGAGCCCTAG |  |
| OsTGA5_Y_Rev | GGATCCTCTTCTTTCGGCCGAGCAAG |  |
| OsTGA2_Bi_For | AGGATCCATGGCAGATGCTAGTTCGAG | BiFC |
| OsTGA2_Bi_Rev | ATGTCGACCTCCCGTGGCCTAGCAAG |  |
| OsTGA3_Bi_For | AGGATCCATGGCTGATACAAGTCCAAG |  |
| OsTGA3_Bi_Rev | ATGTCGACTTCCCTTGGACGGGCG |  |
| OsTGA5_Bi_For | AGGATCCATGGCAGATATGAGCCCTAG |  |
| OsTGA5_Bi_Rev | ATGTCGACTTCTTTCGGCCGAGCAAG |  |
| OsNH1_Bi_For | AGGTACCATGCCGGCGCGTAG |  |
| OsNH1_Bi_Rev | TGGATCCTCATTTCTTTGCAACCTTGGG |  |
| OsNH2_Bi_For | AGGTACCATGGAGCCGCCGACC |  |
| OsNH2_Bi_Rev | TGGATCCTCATCTCCTTGGTCGAATG |  |
| OsNH3_Bi_For | AGGTACCATGGAGACGTCCACCATAAG |  |
| OsNH3_Bi_Rev | TGGATCCTTACCGTGATAGCTTCCCTTTC |  |
| OsNH4_Bi_For | AGGTACCATGGAGGAAACCCTCAAGTC |  |
| OsNH4_Bi_Rev | TGGATCCTCAGGGGAAGCCATGTGG |  |
| OsNH5_Bi_For | AGGTACCATGAGCTCCGAGGACTCG |  |
| OsNH5_Bi_Rev | TGGATCCTTATGCGAAGCCATTGGG |  |
| OsTGA2_OX_For | GGGGACAAGTTTGTACAAAAAAGCAGGCT | Overexpressor |
| OsTGA2_OX_Rev | GGGGACCACTTTGTACAAGAAAGCTGGGT |  |
